# Are diverse societies less cohesive? Testing contact and mediated contact theories

**DOI:** 10.1371/journal.pone.0193337

**Published:** 2018-03-29

**Authors:** Sarah McKenna, Eunro Lee, Kathleen A. Klik, Andrew Markus, Miles Hewstone, Katherine J. Reynolds

**Affiliations:** 1 Research School of Psychology, The Australian National University, Canberra, ACT, Australia; 2 School of Psychological and Clinical Sciences, Charles Darwin University, Casuarina, NT, Australia; 3 School of Philosophical, Historical and International Studies, Monash University, Caulfield, VIC, Australia; 4 Oxford Centre for the Study of Intergroup Conflict, Department of Experimental Psychology, University of Oxford, Oxford, Oxfordshire, United Kingdom; Institut Català de Paleoecologia Humana i Evolució Social (IPHES), SPAIN

## Abstract

Previous research has demonstrated that there is a negative relationship between ethnic diversity in a local community and social cohesion. Often the way social cohesion is assessed, though, varies across studies and only some aspects of the construct are included (e.g., trust). The current research explores the relationship between diversity and social cohesion across a number of indicators of social cohesion including neighbourhood social capital, safety, belonging, generalized trust, and volunteering. Furthermore, social psychological theories concerning the role of positive contact and its impact on feelings of threat are investigated. Using a sample of 1070 third generation ‘majority’ Australians and structural equation modelling (SEM), findings suggest ethnic diversity is related to positive intergroup contact, and that contact showed beneficial impacts for some indicators of social cohesion both directly and indirectly through reducing perceived threat. When interethnic contact and perceived threat are included in the model there is no direct negative effect between diversity and social cohesion. The theoretical implications of these findings are outlined including the importance of facilitating opportunities for positive contact in diverse communities.

## Introduction

Across Europe and North America, research has found that ethnic diversity is detrimental to a range of social cohesion indictors, most commonly trust and volunteering [1]. These findings are concerning given the high rates of immigration characterizing almost every modern society. Particularly amongst members of majority ethnic groups, it is argued that ethnic diversity causes people to withdraw from society in general [1]. Moreover, people living in ethnically heterogeneous neighbourhoods perceive a greater threat to resources (such as jobs) and to their way of life, which also negatively impacts social cohesion. Conversely, research also suggests that immigration affords positive contact experiences between members of different ethnic groups leading to cooperation and respect, which can improve social cohesion [2]. Given these competing theoretical frameworks and findings, it is imperative that further research is conducted in order to gain a clearer understanding of the relationship between ethnic diversity and social cohesion.

The current research investigates whether the diversity and social cohesion relationship highlighted by Putnam’s [1] seminal work leads people to withdraw from social life. Also examined are the role of perceived threat from minority groups and intergroup contact (contact theory [3]), particularly when it also reduces threat (mediated contact theory [2]). This work is important as it is the first study of its kind using an Australian sample and offers significant advancement on previous work. The significance and innovation includes 1) a test of the role of threat and intergroup contact in helping to explain the relationship between ethnic diversity and social cohesion; 2) the inclusion of a comprehensive range of indicators of social cohesion (i.e., generalized trust, volunteering, neighbourhood social capital, safety and belonging) [4]; 3) using an Australian sample where as a multicultural nation there are more opportunities for intergroup contact; and 4) using a total effects analysis to assess whether the relationship between diversity and social cohesion is in fact negative when all of the relevant theoretical constructs are included in the one model [5]. Before the models are outlined in detail, key constructs are defined and support or otherwise for the existing theoretical frameworks are described.

### Defining ethnic diversity and social cohesion

*Ethnic diversity* can be understood in a number of different ways, including as the likelihood that two randomly selected people from the same neighbourhood will have different nationalities (ethnic fractionalization) or as the proportion of immigrants within a particular neighbourhood (relative group size) [6]. Fractionalization has been criticized for being “color-blind”, as a community with 80% whites and 20% blacks has the same degree of ethnic diversity as a community with 20% whites and 80% blacks; for this reason relative group size is preferred. Ethnicity can also be defined using a range of characteristics including physical presentation, language, citizenship or country of origin [7]. The measurement of ethnic diversity varies between studies, however there is a plethora of research to support the use of linguistic measurements, as language is both an objective and salient feature of ethnicity [6, 8–12]. Thus, in the current research a measure of relative group size and ethnicity defined by language are used.

There is also variability in the definition and measurement of social cohesion [1, 4, 13]. Schiefer and van der Noll [4] define social cohesion as “a descriptive attribute of a collective, indicating the quality of collective togetherness” (p.17). Three characteristics can be used to evaluate the quality of togetherness within a given community; 1) social relations (the quality and quantity of people’s relationships with other members of their community); 2) attachment or belonging (meaning identification with the social unit to which you belong); and 3) orientation towards the common goal (including feelings of responsibility for the common good and willingness to comply to social rules and norms).

Having defined the key constructs, the research regarding the relationship between ethnic diversity and social cohesion will be outlined in more detail. The main findings are 1) that ethnic diversity is negatively related to social cohesion (ethnic diversity–social cohesion relationship), 2) that perceived threat negatively mediates this relationship (role of threat), enhancing the detrimental effect of diversity and 3) that intergroup contact positively mediates this relationship, both directly (contact theory), as well as, 4) by reducing threat perceptions (mediated contact theory). Each of these patterns are also further investigated in the current research.

### The ethnic diversity and social cohesion relationship

Research suggests that neighbourhoods with high levels of ethnic diversity have correspondingly lower levels of social cohesion [1]. More recently, a large body of research has investigated the correlates of neighbourhood level diversity [1, 14]. In Putnam’s [1] seminal study, almost all indicators of social cohesion (e.g., attitudes to government and media, happiness, number of close friends, time spent watching television, likelihood of volunteering, and trust in others, amongst others) were negatively related to ethnic diversity, the only exception being organisational involvement. No subsequent study has used such a broad range of indicators however, the relationship has been consistently replicated with outcomes including trust, volunteering, and organisational involvement [5, 15].

The finding that ethnic diversity is negatively related to social cohesion could have important social consequences. Before drawing firm conclusions, though, across a number of studies evidence suggests that the effects of ethnic diversity cannot be generalized across different indicators of social cohesion. Such findings raise questions about social cohesion as a unified construct and qualify conclusions concerning the relationship between diversity and social cohesion. Even in Putnam’s [1] study, aspects of social cohesion such as organizational activity (including religious activity) and political engagement were found to be positively (not negatively) related to ethnic diversity. Research in the UK found that whilst ethnic diversity was negatively related to generalized trust, there was no evidence that it impacted on frequency of volunteering or attitudes towards neighbours [14, 16]. The above research suggests that ethnic diversity differentially effects various aspects of social cohesion. These findings raise concerns about much of the research to date that examines the impact of diversity and perceived threat using only one or two dimensions of social cohesion [3, 17–18]. If, as this research suggests, it is not possible to generalize results across measures of social cohesion, then the relationship between ethnic diversity and social cohesion can only be understood by accounting for a diverse range of indicators of social cohesion.

A possible explanation for this discrepancy provided by van der Meer and Tolsma [7] is that the negative effects of heterogeneity are limited to intra-neighbourhood cohesion. Ethnic diversity impacts trust and cooperation between neighbours, but does not negatively impact social cohesion on a country or city level. Research examining ethnic diversity and social cohesion, at the country or city level, has consistently failed to demonstrate a negative relationship; suggesting that this effect can only be found at the neighbourhood level [19–22]. A notable exception is the US, where support for a negative relationship between diversity and social cohesion (referred to as constrict theory where there is withdrawal from one’s own and other ethnic groups [1]) has been found for measures of social cohesion other than intra-neighbourhood cohesion [7]. Causal mechanisms, which could explain these findings, are currently lacking, however working theories concern the salience of ethnic group sizes. In other words, the ethnic profile of your neighbourhood is easier to judge than the ethnic profile of your country, thus the influence of neighbourhood ethnic diversity, on outcomes such as trust and volunteering, is more pronounced.

Additionally, socio-structural and economic factors have been found to be important. For instance, in US samples, the relationship between ethnic diversity and social cohesion becomes non-significant when the effects of segregation (when members of different ethnic groups are isolated from each other) are taken into account [2, 23–24]. As such, although the previous research supports a negative relationship between diversity and social cohesion, there are important caveats and a need for further research.

### Explaining the relationship between diversity and social cohesion

Putnam [1] has found a negative relationship between diversity and social cohesion and using a wide range of indicators. According to Putnam [1] this occurs because in ethnically heterogeneous communities there is increased threat and fear that can lead to a withdrawal from social relationships and community life either for one’s own and other ethnic groups (constrict theory) or other ethnic groups specifically (conflict theory). Stemming from this work [1] the first aim of this paper is to extend previous work by exploring the relationship between ethnic diversity and social cohesion using a wider range of social cohesion indicators.

**Fig 1:**
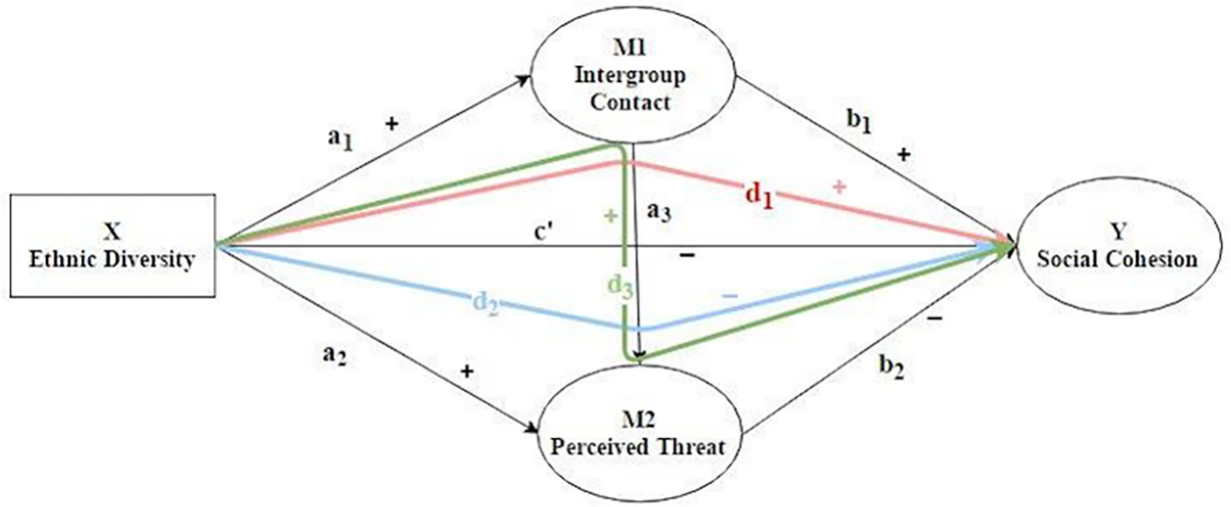
Path diagram. The hypothesized relationship between ethnic diversity and social cohesion as well as the hypothesized mediation effects of intergroup contact and perceived threat.

H1: Ethnic diversity will be negatively related to social cohesion (ethnic diversity–social cohesion relationship). As shown in Fig 1, the relationship from X to Y (path c (c’)) will be negative.

The second aim is to examine if perceived threat negatively mediates the diversity–social cohesion relationship, enhancing the detrimental effect of diversity. The central idea is that ethnic diversity leads to perceived threat (symbolic and realistic) where people fear, hold negative attitudes toward, and withdraw from members of other ethnic groups [24]. *Perceived threat* refers to the belief, held by majority ethnic group members, that their physical well-being and worldview will be threatened, when minority ethnic group members increase in size [24–26]. In a number of studies, threat perceptions amongst members of the majority ethnic group, were positively related to immigration levels, exclusionary attitudes and antisocial behaviours, including prejudice towards minority group members [24, 27–28]. Consequently, it seems that the negative relationship between ethnic diversity and social cohesion is enhanced by the perception, held by majority ethnic groups, that increased ethnic heterogeneity is detrimental to their well-being [24, 27–28].

H2: Threat will negatively mediate the relationship between ethnic diversity and social cohesion (role of threat). As shown in Fig 1, the relationship from X to Y through M2 (path a_2_, path b_2_, and indirect effect path d_2_) will be significantly negative.

More recently, social psychology has applied insights regarding the beneficial effects of intergroup contact, to help explain the relationship between ethnic diversity and social cohesion [2, 29]. *Intergroup contact* refers to interactions between members of different ethnic groups. Not unexpectedly, such interactions become more common when the number of minority group members within a population increases. There is a well-established field of research regarding the potential for intergroup contact to promote positive intergroup outcomes, especially under the optimal conditions of equal status, common goals, intergroup cooperation, and authority support [2, 29]. Much of the research on intergroup contact has focused on the impact of positive contact, such as interethnic friendship, which facilitates these optimal conditions and positive intergroup relations [2, 29]. Recent studies have been able to demonstrate that the effect of positive interethnic friendship and social interactions in heterogeneous neighbourhoods is to improve outgroup attitudes, mitigating the negative effects of increased ethnic diversity [3, 30–31]. Along these lines, the current research employs a measure of interethnic friendship which is commonly used as a proxy for positive intergroup contact.

H3: Intergroup contact will positively mediate the relationship between ethnic diversity and social cohesion (contact theory). As shown in Fig 1, the relationship from X to Y through M1 (path a_2_, path b_2_, and indirect effect path d_1_) will be significantly positive.

Research also suggests that contact impacts social cohesion through a reduction in prejudice; this effect is known as *mediated contact theory* [5, 32]. Thus, intergroup contact can mediate the ethnic diversity and social cohesion relationship in two ways; 1) by directly increasing social cohesion and 2) by reducing threat perceptions. Finally, when all of the direct and indirect effects described above are taken into account, ethnic diversity and social cohesion are no longer negatively related [5]. In a study by Schmid and colleagues [5], when a total effect was calculated (which was the sum of the direct relationship and the three mediation effects), ethnic diversity was not related to intergroup trust or out-group attitudes; although it was significantly negatively related to intragroup trust and neighbourhood trust.

H4: Intergroup contact will negatively mediate the relationship between ethnic diversity and threat and in turn this effect will be positively related to social cohesion (mediated contact theory). As shown in Fig 1, the relationship from X to Y through M1 and M2 (path a_1_, path b_1_, path a_2_, path b_2_, path a_3_, path d_1_, path d_2_ and path d_3_) will be significantly positive in total.

As such, the ethnic diversity and social cohesion relationship cannot be understood without accounting for the mediation effects of negative outgroup attitudes and group contact. The research is important because it integrates the various trajectories of work that have surrounded Putnam’s [1] original study and includes a range of indicators of social cohesion [5, 32].

### The present study

Putnam [1] is the only study to examine the relationship between ethnic diversity and social cohesion using a comprehensive measure of social cohesion. The aim of the present study is to extend previous work by exploring the relationship between ethnic diversity, and social cohesion, threat, and intergroup contact using a wider range of social cohesion indicators. In line with Chan et al. [13], the current study will measure generalized trust, volunteering, neighbourhood social capital, safety and belonging as indicators of social cohesion.

The measure of ethnic diversity used in this study was the proportion of people within a neighbourhood who have immigrated to Australia from a non-English speaking country. It is more common to measure diversity as the likelihood that two randomly selected members of the population will come from different racial groups. Basic categorisations of race (such as Hispanic, non-Hispanic white, non-Hispanic black and Asian) are used which vary between studies. As discussed above, the present study uses a linguistic ethnicity measure because as Fearon’s [9] argues language is objective and easily observable.

Another important aspect of this study is the use of an Australian sample. Two previous studies have been conducted in Australia, but only with respect to trust [33–34]. It has been argued that research regarding social cohesion and social capital has a tradition of being conducted in ethnically homogenous, traditional societies [18]. By contrast, approximately 1 in every 3 people residing in Australia are immigrants (comparatively, approximately 14% of the population residing in the United States are migrants) [35]. By conducting this research in an immigrant nation, namely Australia, the current study addresses this gap and explores how social cohesion works where there are many ethnic groups and more opportunities for intergroup contact.

This study also controlled for a number of variables, including: age, education, employment status, gender and socio-economic status. As discussed above, these variables have been used in past studies and have been shown to be highly correlated with social cohesion [1, 14]. It is, therefore, important to account for the effect of these variables on the relationship between ethnic diversity and social cohesion.

## Methods

### Existing data

This study was approved by Monash University Human Research Ethics Committee, reference number CF07/1240–2007000319. Data used in the present study was collected as part of the 2014 Mapping Social Cohesion study, conducted by the Scanlon Foundation [36]. There have been seven Mapping Social Cohesion reports published since 2007. The aim of these reports is to summarise results from an annual survey that explores the social impact of immigration on Australian society. Each survey builds on the previous year and is used to create the Scanlon Monash Index of Social Cohesion. The 2014 Mapping Social Cohesion report used the same data set as the current study [36].

### Participants

Participants were all born in Australia and both their parents were also born in Australia. The current participants thus consist of only third plus generation immigrants or non-immigrants when immigrants are defined as people born in other countries or whose parents were born overseas [37]. These Australians are the most likely to experience feelings of threat from immigration.

Table 1 displays the demographic information for this sample. There were 1070 Australian participants consisting of 557 males (52.06%) and 513 females (49.74%). This is almost equivalent to the proportion of third plus generation Australians within the general population who are males (49.17%) and females (50.83%) [38]. Given the third generation sample is predominately Anglo-Australians there was no ethnicity demographic question included in the survey. The largest age category was 25–34 (21.40% of participants) [38].

**Table 1:** Sample characteristics.

Population Demographic	*N (%)*
*Gender*	
Male	557 (52.06%)
Female	513 (49.74%)
*State*	
New South Wales	361 (33.70%)
Victoria	293 (27.40%)
Queensland	222 (20.70%)
South Australia	92 (8.60%)
Western Australia	51 (4.77%)
Tasmania	28 (2.62%)
Australian Capital Territory	20 (1.90%)
Northern Territory	3 (0.30%)
*Age*	
18–24	119 (11.12%)
25–34	229 (21.4%)
35–44	179 (16.73%)
45–54	181 (16.92%)
55–64	177 (16.54%)
65–74	131 (12.24%)
75 or over	54 (5.05%)
*Education*	
Up to and including Year 12	334 (31.2%)
Trade or Diploma	368 (34.4%)
Bachelor’s degree or higher	361 (33.7%)
*Employment Status*	
Employed	608 (56.8%)
Retired	241 (22.5%)
Home Duties	87 (8.1%)
Students	80 (7.5%)
Unemployed	55 (5.1%)
*Financial Situation*	
Prosperous or very comfortable	120 (11.3%)
Reasonably comfortable	401 (37.5%)
Just getting along	387 (36.2%)
Struggling to pay bills or poor	144 (13.5%)

### Materials and procedure

Participants were recruited online and invited to participate directly via email. Respondents were incentivised 300 points (equivalent to $3.00 AUD) for their participation in this survey. The survey included a range of measures and administrative data concerning diversity levels and demographic backgrounds. Socio-economic indicators for neighbourhoods were available from the Australian Bureau of Statistics [39].

Of the total 80 items in the Scanlon Mapping Social Cohesion survey, the current study analysed 21 in order to test the proposed hypotheses. Each measure that was modelled in the current study is described below (see all items S1 File). Where possible, all psychological constructs of threat and social cohesion sub-factors were examined with confirmatory factor analysis (CFA) in order to test whether the items validly indicate the respective latent construct. Reliability analysis results are presented below in the current section.

#### Intergroup contact: Mediator 1

Intergroup contact was construed using two behavioural items (*α* = .74). Both items (e.g., “Overall, approximately how many of your friends are from other cultures?” and “How many of your best friends are from other cultures?”) concerned quantitative interethnic friendships. Participants reported how many of their friends are from other cultures. There were 11 possible responses from “none” to “more than 10” in the original survey. These items were recoded into a 1–7 scale from “no friends” to “more than 5”. This scale could be used as a continuous variable rather than a truncated count variable, which was preferable, as well as being more reflective of other intergroup contact measures used in the existing literature [40].

#### Threat: Mediator 2

Threat was measured using five items (*α* = .81). Four of the items (e.g., “Do you feel positive, negative or neutral about the following categories of people coming to living in Australia as permanent or long-term residents?”—(1) “Skilled workers [e.g., doctors or nurses, plumbers etc.]”, (2) “Those who have close family living in Australia [i.e., parents or children]”, (3) “Refugees who have been assessed overseas and found to be victims of persecutions and in need of help”, and “Young people who want to study in Australia”) were on five-point scales ranging from “strongly positive” to “strongly negative”. The fifth item (“What do you think of the number of immigrants accepted into Australia at present? Would you say it is…?”) was on a three-point scale from “too low” to “too high” and was reverse coded so that a higher score would reflect a greater threat perception. All items were then z-scored before creating the threat measure.

#### Social cohesion sub-factors: Outcome variables

Consideration of item content as well as a review of the literature suggested that 11 of the items provided in the Mapping Social Cohesion survey should be used in the present study to measure social cohesion. These items were theorized to belong to three distinct components, which were named neighbourhood social capital, safety, and belonging. In all cases where there was variability in response formats, items were turned into z-scores before forming scales.

1. The first factor, neighbourhood social capital consisted of four items. The first three items (e.g., “People in my local area are willing to help their neighbours?”, “My local area is a place where people from different national or ethnic backgrounds get on well together.”, “I am able to have a real say on issues that are important to me in my local area.”) were on a five-point scale from “strongly agree” to “strongly disagree”. The fourth item (e.g., “Would you say that living in your local area is becoming better or worse, or is it unchanging?”) was on a five-point scale from “much better” to “much worse”. These items measured the amount of social capital (including informal help and social ties) which participants thought they had within their local area (*α* = .77). Items were reverse coded so that higher scores reflected more perceived neighborhood social capital within their local area.
2. The second factor, safety, was comprised of two items. The first item (“How safe do you feel walking alone at night in your local area?”) was originally reported on an eight-point scale, however four of the possible responses (“neither safe nor unsafe”, “never walk alone at night”, “didn’t know” and “preferred not to answer”) made up less than five percent of the total responses and were not appropriate for a Likert-type scale. Thus, the first item was recoded to a four-point scale from “very safe” to “very unsafe” and all other responses were treated as missing data. The second item (“Thinking about all types of crime in general, how worried are you about becoming a victim of crime in your local areas?”) was on a four-point scale from “very worried” to “not worried at all”. Together, these items were thought to reflect participants’ beliefs about the safety of their local area (α = .77). Items were reverse coded so that a higher score reflected a greater feeling of safety.
3. The belonging factor was comprised of three items. The first two items (“To what extent do you take pride in the Australian way of life and culture?” and “To what extent do you have a sense of belonging in Australia?”) were on four point scales from “to a great extent” to “not at all”. The third item (“In the modern world, maintaining the Australian way of life and culture is important.”) was on a five point scale from “strongly agree” to “strongly disagree”. These items were thought to reflect whether the Australian way of life was important to participants and was named belonging (*α =* .79). Items were reverse coded so that perceiving the Australian way of life as important was reflected by a high score.

The remaining measures include 4) one item measuring generalized trust (“Generally speaking would you say that people can be trusted or that you can’t be too careful?”, using a three point scale from “can’t be too careful” to “can be trusted”) and 5) one item measuring volunteering (“How often do you participate in voluntary activity?”, using a five point scale from “less than once a year” to “at least once a week”) were also included in the analysis. The generalized trust measure has been widely used [18. 31] and represents an important indicator of social cohesion [13].

#### Diversity index

A diversity index [39] using 2011 census data was merged to the survey data set and used as an independent variable. Diversity is the proportion of people residing in each postcode who were born either in Australia or overseas in an English-speaking country. English-speaking countries were limited to the UK, Ireland, US, Canada, New Zealand, and South Africa. The formula used to calculate the diversity score for each postcode was (1 - (number of residents born overseas in a non-English speaking country ÷ total number of residents)). This item was reverse coded in the current study, so that higher scores would be indicative of greater ethnic diversity.

#### Socioeconomic Indexes for Areas (SIEFA)

SEIFA scores were calculated using 2011 census data by the Australian Bureau of Statistics (ABS) [39]. SEIFA combines four indexes that have been created from social and economic census data. A score for a Statistical Area Level 1 (SA1) is created by adding together the weighted characteristics of that SA1 and standardizing the total (mean = 1000, standard deviation = 100; [41]). A lower score indicates that an area is relatively disadvantaged compared to an area with a higher score.

#### Demographic covariates

Based on previous research discussed above, it was deemed necessary to control for a number of individual and socio-economic factors including gender, age, employment status, education, financial status and the SEIFA score of the participants’ local area [1, 14].

#### Analytical strategy

Multilevel analysis was not necessary even though the participants were nested in postcodes because the mean number of participants in each postcode was 1.81 so design effects were negligible [42]. The main SEM analysis using Mplus included both observed and latent variables. Fig 1 shows the hypothesized paths between diversity, intergroup contact, perceived threat, and social cohesion. The three social cohesion factors, as well as items measuring trust and volunteering, were tested one by one as outcome variables. Each of the social cohesion and perceived threat factor variables were examined as a measurement model (i.e. as a latent variable with item loadings). Modification indices were used during the main SEM analysis to improve model fit. Correlations between unknown common random errors among some items (only items belonging to the same factor) were estimated, rather than assuming that they were fixed at zero, when the residual correlations were deemed large and made theoretical sense.

A series of hierarchical and contrasting SEMs were conducted for each of the social cohesion outcomes. Model 1 examined the impact of six demographic covariates on social cohesion. Model 2 included the independent variable of ethnic diversity in addition to the covariates in explaining social cohesion. Model 3 further included perceived threat as another explanatory variable, whereas Model 4 examined the mediation effect of threat in the relationship between diversity and social cohesion with all covariates controlled. Model 5 added contact as another explanatory variable of social cohesion and subsequently Model 6 tested contact theory, which concerned whether contact mediates the impact of diversity on social cohesion when demographic variables are controlled. Model 7 tested mediated contact theory which required estimating three indirect effects. In addition to the mediation effects of threat and contact, described above, this model tested whether contact mediates the relationship of ethnic diversity to threat and the effect of this mediation on its subsequent relationship to social cohesion.

## Results

### 1. Data screening

Prior to main analysis, variables were screened for missing data, normality, outliers and the assumptions of multivariate normality. For the main SEMs, the maximum likelihood (ML) method of expectation maximization (EM) was used to impute missing data [43]. Compared to traditional regression methods, EM provides a less biased set of imputed values [44]. In order to account for non-normality of variables, the ML estimator with robust standard errors (MLR) was selected with MPlus coding for the model coefficients [45]. Finally, inspection of bivariate scatterplots confirmed the linearity and homoscedasticity of the data. Table 2 displays the inter-variable correlations for the variables of interest.

**Table 2:** Inter-variable correlations (*N* = 1040).

	1	2	3	4	5	6	7	8	9	10	11	12	13	14	15	16
1.Diversity	1															
2.Gender	-.063*	1														
3.Age	-.172**	-0.014	1													
4.Empl.	.115**	-.070*	-.378**	1												
5.Retired	-.140**	-0.053	.651**	-.618**	1											
6.Student	0.019	.166**	-.204**	-.494**	-.232**	1										
7.Educ.	.189**	-0.006	-.288**	.231**	-.195**	-.070*	1									
8.Income	.075*	-0.059	0.023	.108**	0.047	-.094**	.154**	1								
9.SEIFA	.080**	0.001	-.074*	0.036	-0.037	0.028	.163**	.122**	1							
10.Trust	.078*	-0.031	0.050	-0.021	0.049	-0.012	.126**	.109**	.064*	1						
11.Volunt.	-.078*	0.002	.172**	-.145**	.170**	-0.027	0.041	0.053	0.022	.111**	1					
12.Safety	0.017	-.110**	-0.012	0.006	0.008	-0.006	0.039	.086**	0.038	.061*	0.005	1				
13.Neigh.Cap.	-0.018	0.041	.099**	0.004	0.055	-0.048	.091**	.174**	.081**	.176**	.113**	.085**	1			
14.Belon	-.067*	0.041	.332**	-0.055	.242**	-.139**	-0.049	0.047	-0.026	0.057	.139**	0.011	.179**	1		
15.Cont.	.111**	0.059	-0.039	.091**	-.093**	-0.021	.232**	0.048	.145**	.194**	.149**	0.033	.272**	.062*	1	
16.Neg. Grp.	-0.053	-0.036	0.003	0.020	-0.036	-0.001	-.219**	-.090**	-.109**	-.260**	-.084**	-.069*	-.275**	0.049	-.375**	1
$\bar{x}$												11.17	5.42	10.18	9.79	12.23
*SD*												3.61	1.41	2.27	3.31	3.80

*Correlation is significant at the 0.05 level (2-tailed)

**Correlation is significant at the 0.01 level (2-tailed); 1.Ethnic Diversity of Australian postcode areas estimated by ABS [41]; 2. % of males coded as 1 compared to females coded as 2; 3. Age coded as 1 = 18–24, 2 = 25–34, 3 = 35–44, 4 = 45–54, 5 = 55–64, 6 = 65–74, 7 = 75 or over; 4.Employment status, employed coded as 1 compared to reference group (unemployed and home duties) coded as 0; 5.Retired coded as 1 compared to reference group coded as 0; 6.Students coded as 1 compared to reference group coded as 0; 7. Level of education coded as 0 = up to and including Year 12 and Trade or Diploma, 1 = Bachelor’s degree or higher; 8. Financial status, 1 = prosperous or very comfortable, 2 = reasonably comfortable, 3 = just getting along, 4 = struggling to pay bills or poor; 9.Socioeconomic Indexes for Areas [41]; 10. Generalized Trust; 11. Volunteering; 12. Safety felt in the neighbourhood 13. Neighbourhood Social Capital; 14. Belonging; 15. Intergroup Contact; 16. Negative Outgroup Attitudes.

The interscale correlation matrix for all variables is presented in Table 3 including the control variables gender, age, employment, finances, education and SEIFA index. There was no evidence of multicollinearity or singularity, which occurs when variables are correlated too highly (.90 and above) [44].

**Table 3:** Direct and indirect standardized coefficients explaining generalized trust, volunteering and social capital (*N* = 1090).

Variables and Paths	Trust			Volunteering			Neighbourhood Social Capital		
	*ß*^a^	*s*.*e*.^b^	95% CI^c^	*ß*	*s*.*e*.	95% CI	*ß*	*s*.*e*.	95% CI
Age^d^	.10*	.04	(.01, .18)	.10*	.04	(.01, .19)	.15**	.05	(.06, .23)
Education^e^	.13*	.04	(.01, .26)	.10**	.03	(.04, .17)	.11**	.04	(.04, .18)
Employed^f^	-.02	.06	(-.22, .18)	-.19*	.08	(-.34, -.04)	.01	.07	(-.11, .15)
Retired^g^	-.02	.06	(-.26, .23)	-.02	.08	(-.16, .13)	-.03	.07	(-.15, .11)
Students^h^	.05	.05	(-.18, .27)	-.09	.06	(-.21, .04)	.01	.06	(-.10, .12)
Income^i^	.06	.03	(.01, .12)	.05	.03	(-.01, .11)	.20**	.04	(.13, .28)
Gender^j^	-.07	.03	(-.17, .04)	.01	.03	(-.05, .08)	.06	.04	(-.01, .13)
SEIFA^k^	.00	.04	(-.00, .00)	.02	.04	(-.06, .11)	.07	.05	(-.02, .16)
Ethnic Diversity	.07	.03	(.03, .71)	-.06*	.37	(-1.23, -.01)	-.07	.04	(-.13, .04)
Ethnic Diversity → Threat	.01	.01	(-.01, .03)	.00	.00	(-.00, .01)	.01	.01	(-.01, .04)
Ethnic Diversity → Contact	.02	.01	(-.00, .04)	.02*	.01	(.00, .03)	.07**	.01	(.05, .10)
Ethnic Diversity → Contact → Threat	.02**	.00	(.01, .02)	.01	.01	(-.00, .01)	.02**	.04	(.01, .03)
Total	.08**	.08	(.02, .14)	-.05	.03	(-.11, .01)	-.01	.04	(-.09, .08)
**Model Fit Indices**^**l**^									
*χ2/(df)*	4.21**			4.01**			3.23**		
*RMSEA*	0.06			0.06			0.05		
*CFI*	0.91			0.91			0.92		
*SRMR*	0.06			0.06			0.06		
*R*^*2*^	0.09**			0.10**			0.21**		

*Significant at the *p <* .*05* level

**Significant at the *p <* .01 level.

a. standardized beta, direct and indirect coefficients of the variables

b. standard error estimates associated with standardized coefficients

c. 95% confidence intervals associated with standardized coefficient estimates

d. Age coded as 1 = 18–24, 2 = 25–34, 3 = 35–44, 4 = 45–54, 5 = 55–64, 6 = 65–74, 7 = 75 or over

e. Level of education coded as 0 = up to an including Year 12 and Trade or Diploma, 1 = Bachelor’s degree or higher

f. Employment status, employed coded as 1 compared to reference group (unemployed and home duties) coded as 0

g. Retired coded as 1 compared to reference group coded as 0

h. Students coded as 1 compared to reference group coded as 0

i. financial status 1 = prosperous or very comfortable, 2 = reasonably comfortable, 3 = just getting along, 4 = struggling to pay bills or poor

j. % of males coded as 1 compared to females coded as 2

k. Socioeconomic Indexes for Areas (SIEFA)

l. fit indices associated with model

Reliability analysis was performed for the measures of perceived threat, intergroup contact and social cohesion. As detailed above, Cronbach-Alpha levels ranged from .74 to .81 suggesting acceptable internal consistency [44]. In addition, all corrected item-total correlations were above .30 indicating that items correlate to an acceptable degree with other items in the scale.

### 2. Main analysis: Structural Equations Modelling (SEM)

The measurement models and path analysis using SEM allowed testing of all of the direct and indirect relationships between ethnic diversity and social cohesion. There were five social cohesion measures thus six groups of hierarchical models were run in total. As displayed in Tables 3 and 4, in all cases, the *χ2* statistic was significant which suggested poor model fit. However, large sample sizes will cause *χ2* to be significant even when there is no significant difference between the observed and predicted correlation matrices [46]. For this reason, other fit indices were considered, including relative *χ2*, RMSEA, CFI and SRMR. Each of the eight models had acceptable model fit indices suggesting the theorized models fit the observed data.

**Table 4:** Direct and indirect standardized coefficients explaining safety and belonging (*N* = 1090).

Variables and Paths	Safety			Belonging		
	*ß* ^a^	*s*.*e*.^b^	95% CI^c^	*ß*	*s*.*e*.	95% CI
Age^d^	.02	.05	(-.07, .11)	.45**	.05	(.10, .17)
Education^e^	.13	.04	(-.06, .09)	.06	.04	(-.02, .16)
Employed^f^	.08	.08	(-.11, .23)	.31**	.10	(.15, .52)
Retired^g^	.10	.07	(-.04, .25)	.25**	.09	(.10, .52)
Students^h^	.04	.06	(-.08, .16)	.16*	.08	(.03, .44)
Income^i^	.24**	.04	(.17, .32)	.03	.04	(-.03, .06)
Gender^j^	-.19**	.03	(-.26, -.13)	.07*	.04	(.00, .15)
SEIFA^k^	.09*	.04	(.00, .15)	-.01	.03	(-.00, .00)
Ethnic Diversity	-.07*	.03	(-.03, .05)	-.02	.03	(-.09, .04)
Ethnic Diversity → Threat	.01	.01	(-.01, .03)	.00	.00	(-.00, .01)
Ethnic Diversity → Contact	.01	.01	(-.01, .03)	.03*	.01	(.00, .05)
Ethnic Diversity → Contact → Threat	.01**	.00	(.00, .01)	.01	.01	(-.00, .01)
Total	-.06	.03	(-.12, .01)	-.02	.21	(-.08, .06)
**Model Fit Indices**^**l**^						
*χ2\(df)*	4.08**			4.07**		
*RMSEA*	0.06			0.06		
*CFI*	0.91			0.91		
*SRMR*	0.06			0.06		
*R*^*2*^	0.17**			0.25**		

* = Significant at the *p <* .*05* level

** = Significant at the *p <* .01 level.

a. standardized beta, direct and indirect coefficients of the variables and paths

b. standard error estimates associated with standardized coefficients

c. 95% confidence intervals associated with standardized coefficient estimates

d. Age coded as 1 = 18–24, 2 = 25–34, 3 = 35–44, 4 = 45–54, 5 = 55–64, 6 = 65–74, 7 = 75 or over

e. Level of education coded as 0 = up to an including Year 12 and Trade or Diploma, 1 = Bachelor’s degree or higher

f. Employment status, employed coded as 1 compared to reference group (unemployed and home duties) coded as 0

g. Retired coded as 1 compared to reference group coded as 0

h. Students coded as 1 compared to reference group coded as 0

i. financial status 1 = prosperous or very comfortable, 2 = reasonably comfortable, 3 = just getting along, 4 = struggling to pay bills or poor

j. % of males coded as 1 compared to females coded as 2

k. Socioeconomic Indexes for Areas (SIEFA)

l. fit indices associated with model

Explained variance (*R*^*2*^) was also calculated for each measure. As can be seen in Tables 3 and 4, Model 7 accounted for around 20% of total variance in the case of neighbourhood social capital, safety and belonging. Also, Model 7 accounted for around 10% of the variance in volunteering and generalized trust.

Post-Hoc power analysis showed that, with a sample size of 1070, power exceeded .99 for each of these effect sizes. Large sample size gave this study high statistical power, thus both significance level and effect size should be considered when interpreting results.

#### Generalized trust

Generalized trust was significantly positively related to age (*ß* = .10, *p* = .016) and education (*ß* = .13, *p* < .001). Table 3 shows Model 7 with all effects examined. Older people and those who were more highly educated, reported higher levels of generalized trust. Ethnic diversity was not directly related to generalized trust (H1).

The indirect effect of diversity on generalized trust via contact, was not significant, and neither was the indirect effect via threat (H2; H3). However, the indirect effect via contact and threat was significantly positive (H4; *ß* = .02, *p* < .001). More diversity was associated with more contact and more contact was associated with less threat which led to more generalized trust. The total relationship between ethnic diversity and generalized trust was significantly positive (*ß* = .08, *p* = .014) meaning that, when all the effects of contact and threat were taken into account ethnic diversity was associated with higher levels of generalized trust.

#### Volunteering

As shown in Table 3, volunteering was significantly positively related to age (*ß* = .10, *p* < .029), education (*ß* = .07, *p* = .035), and employment status (*ß* = -.19, *p* < .05). Those who volunteer are more likely to be either older, more highly educated, or unemployed.

Volunteering was significantly negatively related to diversity (H1; *ß* = -.06, *p* = .034) meaning that increases in ethnic diversity were associated with decreases in frequency of volunteering. Intergroup contact significantly mediated the relationship between ethnic diversity and volunteering (H2; *ß* = .02, p = .037). The increase in intergroup contact which accompanied ethnic diversity led to greater frequency of volunteering. No other mediation effects were significant (H3; H4). The total relationship between ethnic diversity and volunteering was not significant.

#### Neighbourhood social capital

Neighbourhood social capital was significantly positively related to age (*ß* = .15, *p* < .001), education (*ß =* .11, *p <* .001), and income (*ß* = .20, *p* < .001). Older, more educated people, and those who were financial security had more positive views of their communities (see Table 3). Neighbourhood social capital was not significantly related to ethnic diversity (H1). The indirect effect via contact was significantly positive (H3; *ß* = .07, *p* < .001) as was the indirect effect via contact and threat (H4; *ß =* .02, *p* < .001). This finding suggests that neighbourhoods higher in ethnic diversity experienced more intergroup contact which was related to greater neighbourhood social capital partly because of reduced threat perceptions. No other indirect effects were significant (H2). The total relationship between ethnic diversity and neighbourhood social capital was not significant.

#### Safety

As shown in Table 4, safety was positively related to income (*ß* = .24, *p* < .001) and SEIFA score (*ß* = .09, *p* = .047), suggesting that people who were more financially secure and lived in areas with high socio-economic status believed their neighbourhoods were safer. Safety was significantly negatively related to gender (*ß =* -.19, *p* < .001) suggesting that men were more likely to report living in a safe area.

Safety was significantly negatively related to ethnic diversity (H1; *ß* = -.07, p = .031) meaning that people who lived in highly diverse neighbourhoods reported feeling less safe than people in neighbourhoods with more ethnic homogeneity, even after controlling for socio-economic factors. Neither intergroup contact nor perceived threat mediated the relationship between ethnic diversity and safety on their own (H2; H3). The indirect effect via contact and threat was found to be significantly positive (H4; *ß* = .01, *p* = .010). The total relationship between ethnic diversity and safety was not significant. This suggests that increases in diversity were associated with increases in contact, and increases in contact with lower threat perceptions, such that increases in diversity did not adversely affect perceived safety.

#### Belonging

Belonging was significantly related to age (*ß* = .45, *p* < .001), gender (*ß =* .07, *p* = .042), being employed (*ß* = .31, *p* = .001), being retired (*ß* = .25, *p* = .007), and being a student (*ß =* .16, *p =* .037), as shown in Table 4. This suggests that older people reported greater belonging than young people and women reported more belonging than men. People who were employed, retired or students reported higher belonging than people who had home duties or other. Belonging was not related to ethnic diversity (H1). Intergroup contact positively mediated the relationship between ethnic diversity and belonging (H3; *ß* = .03, *p* = .028). No other indirect effects were significant (H2; H4). The total relationship between ethnic diversity and belonging was not significant. This suggests that people living in more ethnically heterogeneous areas did not report lower sense of belonging.

## Discussion

This study offers significant advance on previous work; not only because the data could be used to assess the role of threat and intergroup contact on the diversity–social cohesion relationship but also because a more comprehensive range of indicators of social cohesion (i.e., generalized trust, volunteering, neighbourhood social capital, safety, and belonging) are included. Additionally, a total effects analysis was conducted in order to assess whether the diversity and social cohesion relationship is in fact negative when all the constructs (and paths) are included in the one model [5].

More specifically, it was hypothesized that the relationship between ethnic diversity and social cohesion would be negative (H1). It was also hypothesised that threat would negatively mediate the relationship between diversity and social cohesion (H2). Drawing more explicitly on recent social psychological theory and research, it was predicted that intergroup contact would positively mediate the relationship either directly (contact theory; H3) or through perceived threat (mediated contact theory; H4).

### Evaluation of hypothesis

Using an Australian sample there was some evidence to support the notion that ethnic diversity is associated with less social cohesion but only for certain social cohesion indicators (see Table 5 for summary). Ethnic diversity was significantly negatively related to volunteering and safety (H1). Perceived threat did not negatively mediate the relationship between ethnic diversity and the social cohesion variables (H2). Greater ethnic diversity had some negative implications for safety and volunteering, but no other aspect of social cohesion, and this relationship was not related to threat perceptions.

**Table 5:** Summary of support for hypothesis.

	H1	H2	H3	H4	Total effects
Trust	N	N	N	Y	*p* <0.01
Volunteering	Y	N	Y	N	NS
Neighbourhood Social Capital	N	N	Y	Y	NS
Safety	Y	N	N	Y	NS
Belonging	N	N	Y	N	NS

H1: ethnic diversity–social cohesion relationship; H2: role of threat; H3: contact theory; H4; mediated contact theory; Y: yes, hypothesis supported. N: No, hypothesis not supported. NS: Non-significant.

Exploring the role of intergroup contact (contact theory), there was significant mediation for the social cohesion indicators of volunteering, neighbourhood social capital and belonging (see Tables 3 and 4). There was also support for mediated contact theory, as there was evidence of a significant positive effect from ethnic diversity to generalized trust, neighbourhood social capital, and safety, when intergroup contact was included as a mediator of the relationship between ethnic diversity and perceived threat (H3). Together, this suggested that for all the social cohesion variables, the increase in intergroup contact which accompanied increased ethnic diversity, showed beneficial impacts.

Looking at the total relationships (the sum of direct and indirect effects), none of the various social cohesion indicators assessed in the Mapping Social Cohesion survey were negatively related to ethnic diversity (H4; see Table 5). These findings are important because they suggest that ethnic diversity does not have an adverse effect on social cohesion in this Australian sample. Moreover, for generalized trust (which is the most commonly used indicator used to assess social cohesion) ethnic diversity was found to have a *beneficial* impact. The total relationship between ethnic diversity and generalized trust, which took into account the effects of intergroup contact, was significantly positive.

### The role of threat and intergroup contact

The findings of the current study suggest support for constrict theory [1] with respect to volunteering and safety, when contact and threat were not in the model. This conforms to previous research which has shown that these variables are negatively related to neighbourhood ethnic heterogeneity [1, 33]. However, it was also found that ethnic diversity was not directly related to generalized trust, neighbourhood social capital, or belonging which is not in line with some previous research. In Putnam’s [1] work, all of these variables were lower in ethnically heterogeneous neighbourhoods. Several other studies have been able to demonstrate a negative relationship between ethnic diversity and generalised trust [3, 18]. As such, the results of the current study support past research concerning the relationship between diversity and volunteering and safety but in a limited manner because once other mediators are taken into account the relationship becomes non-significant[1, 33]. The lack of a direct negative relationship between ethnic diversity and social cohesion contradicts past findings including the previous research conducted in Australia [3, 19].

#### Role of threat

The present research attempted for the first time to test the perceived threat mediation effect on variables such as generalized trust, belonging, neighbourhood social capital, and safety. Previous research has shown that perceived threat negatively mediated the relationship between ethnic diversity and social cohesion variables, including prejudice and volunteering [24, 28, 40]. The present research found that this was not the case. More research is needed to establish whether unique aspects of this study, including the use of an Australian sample, as well as the measures of threat and social cohesion that were used, can account for this disparity in results.

#### Contact theory

The current finding that intergroup contact positively mediated the relationship between ethnic diversity and social cohesion, is consistent with previous literature [3, 30–31]. For instance, past research has shown that, the increase in intergroup contact, which is associated with greater ethnic diversity, has positive implications for generalized trust, volunteering, and prejudice when contact is positive. These findings are consistent with the current research. Our study showed that intergroup contact positively mediated the relationship of ethnic diversity to neighbourhood social capital and belonging. Overall, the results indicate that the relationship between ethnic diversity and social cohesion is mediated by positive intergroup contact [3, 30–31].

#### Mediated contact theory

Finally, studies have found that the increase in intergroup contact, which accompanies ethnic diversity, significantly reduces perceptions of threat when the contact is positive. Moreover, this reduction can significantly improve generalized trust and reduce negative outgroup attitudes [15, 31, 45]. The present study found support for mediated contact theory for additional indicators of social cohesion, other than generalized trust. The mediated contact effect had positive implications for safety (that is the perceived safety of the participants’ neighbourhood).

Looking at the total effect, with all the core constructs in the model, ethnic diversity was not negatively related to any aspect of social cohesion. This finding contradicts past research [1, 14, 18]. One previous study which tested total effects, found that ethnic diversity was not related to out-group trust and was negatively related to in-group and neighbourhood trust [40]. Extending this work, using an Australian sample and more diverse indicators of social cohesion, the current research found that the relationship between ethnic diversity and most social cohesion variables was significantly positive not negative. Possible theoretical explanations for this finding are explored below.

### Implications for theory

The results of this study have several theoretical implications. First, the findings suggest that the relationship between various aspects of social cohesion is not well understood. Ethnic diversity was differentially related to the various indicators of social cohesion, meaning that its effects could not be generalized. Previous research has been limited to a relatively small number of social cohesion indicators, including trust and organizational involvement [30, 40]. The results of the present study suggest that this approach has not produced a comprehensive understanding of the relationship between ethnic diversity and social cohesion.

This current research has also contributed to theoretical understandings of the ethnic diversity–social cohesion relationship by further exploring the effect of positive intergroup contact. Few studies have incorporated the key social psychological construct of positive contact when examining the diversity and social cohesion relationship. The results indicate that positive contact between groups can lead to respect and liking between members of the different ethnic groups, meaning that people are less likely to withdraw from society. Although ethnic diversity can have some detrimental effects, these are mitigated by the increased opportunities such diversity creates for contact between ethnic groups and the associated benefits when intergroup contact is positive.

On the basis of this research it is difficult to draw firm conclusions about the relationship between ethnic diversity and social cohesion mainly because the patterns varied depending on the indicator of social cohesion (safety, neighbourhood social capital, belonging, generalized trust and volunteering). What is clear however, is that the role of positive contact in helping to explain this relationship has been undervalued by previous work, including Putnam’s [1] seminal study, creating a falsely negative view of ethnically heterogeneous neighbourhoods. This research calls for a more nuanced understanding of the ethnic diversity and social cohesion relationship, such that future research should identify what settings are optimal for creating positive contact experiences and how interventions in heterogeneous communities can be used to create more intergroup contact and therefore, improve social cohesion outcomes.

### Limitations and suggestions for future research

Despite the important insights provided by this research, there were several limitations to its design which need to be acknowledged. First, factor analysis is weakened when three or fewer items load on a factor [46–47]. In the present study intergroup contact, neighbourhood social capital, safety, and belonging were composed of less than three items. A smaller number of items per factor (*p/f*) inflates standard errors. Additionally, when factors include less than three items, the increase in efficiency is negligible [46–47]. The current measurement models were weakened by latent variables which only had two or three items. In addition generalized trust and volunteering were assessed using one item. Measures of sub-group trust towards one’s own ethnic group and different ethnic groups were not included in the original survey. Such measures would have enabled a more direct and systematic analysis of Putnam’s [1] main theoretical arguments with respect to constrict theory and conflict theory. Generalized trust was used as an indicator of social withdraw from others but more specific measures would have been informative. Future studies need to strengthen measurement of social cohesion by developing more items and valid indicators.

The present study may have failed to reproduce the perceived threat mediation effect due to the specific measure employed. Wording of perceived threat items in past research has directly addressed threat perceptions, for instance “people from ethnic minority backgrounds threaten White British people” or “foreigners living here threaten my way of life” [28, 40]. Items used in this study, though, asked how positively or negatively respondents felt towards immigrants, as well as whether they felt the number of immigrants was too high. These items are more indirect measures of threat and do not specify sources of perceived threat. Again, this study calls for future research to test conflict theory with more comprehensive measures of perceived threat.

The present study measured a proxy for positive intergroup contact–interethnic friendship, but we did not assess how negative intergroup contact impacts the relationship between ethnic diversity and social cohesion. The conclusions we draw relative to intergroup contact is specific to the role of *positive* contact and cannot be generalized to contact experiences more broadly. For example, research suggests that negative intergroup contact is associated with prejudicial attitudes and can be detrimental for positive intergroup outcomes [48–49]. The work on negative intergroup contact highlights that contact can be experienced in a variety of ways, which can differ in intensity, frequency, and proximity and can be positive and/or negative [49–52]. Also recent research suggest that negative contact experiences may increase prejudice to a greater extent than positive contact decreases it [49]. Thus, it is plausible that positive and negative intergroup contact differentially impact on the ethnic diversity–social cohesion relationship, but more research is needed to expand upon the measurement of intergroup contact employed in the current study. As of yet, no research has compared the mediation effects of positive and negative intergroup contact.

A further limitation of the present study was that ethnicity was not examined with respect to different groups [53–55]. It should be noted that there are marked differences in patterns of contact with and attitudes towards different ethnic groups. For instance, previous research in Australia found that participants held more negative attitudes towards immigrants who were Arabic and Lebanese than immigrants from other non-English speaking countries such as Vietnam and China [54]. Future research is needed to establish whether these differences in attitude have a significant impact on levels of intergroup contact or perceived threat and subsequently on the ethnic diversity and social cohesion relationship.

The linguistic measure of ethnic diversity may limit generalisability of results. In Australia, the formal diversity index by Australian Bureau of Statistics (ABS) is based on the birth country and the criterion is English speaking [40]. In future work, other ways to assess diversity should be considered. Further, the measurement used in this work may not be appropriate for other countries where ethnic or immigrant status may be a more appropriate indicator of diversity. The diversity measure used in the current research was restricted to countries with a colonial history tied to the UK (i.e., UK, Ireland, USA, Canada, New Zealand, and South Africa) but yet these countries still have potential cultural differences from Australia, which have not been fully explored. Future work could explore foreign country of birth as a diversity measure rather than language. It is unclear how to assess countries for cultural distance (being born in the UK compared to Egypt) from the host nation which also is a variable of interest.

Finally, recent data suggests that ethnic segmentation in Australian cities is increasing; partially because people who are not happy with increasing ethnic diversity are moving away [55–56]. This trend and possible implications were not captured by the present study. As discussed above, previous research in America has found that controlling for segregation accounted for a large part of the negative relationship between ethnic diversity and social cohesion [22–23]. Longitudinal research is needed in order to establish whether this trend will change the nature of the relationship between ethnic diversity and social cohesion in Australian population samples.

The results of this study emphasize future directions for refining measurement of social cohesion. As discussed above, the effects of ethnic diversity were not generalized across measures of social cohesion, therefore in future work it is necessary to go beyond the conventional variables (which are widely used in national surveys) of trust, organizational involvement, and outgroup attitudes. It is also necessary to consider through scale development whether there is a general social cohesion construct that incorporates a range of sub-factors. For this to be achieved, further work will be needed on the operational definition of social cohesion and its measurement. Finally, longitudinal research is necessary in order to address uncertainty about the causal directions of the relationship between ethnic diversity and social cohesion as well as of that between intergroup contact and perceived threat. It is likely that there is an intersection between positive intergroup contact and social cohesion, where these constructs are likely reciprocally related. This would also be a fruitful direction for future research. The present study, did not allow us to make any causal inferences about the nature of the relationships being tested; a limitation which is shared by past research in this field.

### Conclusion

Given the high levels of immigration in almost all modern societies, research showing that ethnic diversity can negatively impact social cohesion, is deeply concerning. Our research shows that this concern is not well founded. The results of the current research indicate that when all the key constructs are included in the models (i.e., diversity, positive contact, perceived threat and social cohesion indicators), ethnic diversity does not have a detrimental effect on any social cohesion variable. Ethnic diversity was positively related to generalized trust through the mediators of intergroup contact and perceived threat. This current research highlights the importance of accounting for the effects of intergroup contact, including its simple mediation effect and its complex mediation effect through perceived threat. The results suggest that further understanding the intergroup contact experience and how to facilitate positive contact, are important for building stronger cohesive communities.

## Supporting information

S1 File Technical report. Survey items.
(PDF)

S2 File SPSS data set. Data set in SPSS.
(SAV)

S3 File Excel data set. Data set in Excel.
(XLSX)
