# Supplementary material for: Are diverse societies less cohesive? Testing contact and mediated contact theories
Source: PLoS One. 2018 Mar 29;13(3):e0193337. doi: 10.1371/journal.pone.0193337 (PMC5875770; doi:10.1371/journal.pone.0193337)
Supplement: S1 File — Survey items. (PDF) [file pone.0193337.s001.pdf]

# Methodology report

002437
Social Cohesion Survey

| | |
|---|---|
| **prepared for:** | **Andrew Markus** |
| | Monash University |
| **your source contact:** | Damien Marquez |
| | Account Manager |
| **tel:** | +61 (2) 9568 8374 |
| **email:** | damien.marquez@yrsource.com |
| **issue date:** | 27-Oct-14 |

# Table of contents


1. Project scope and overview ....................................................................3
2. Timings ..................................................................................................4
3. Sample frame...........................................................................................5
4. Sample size .............................................................................................6
5. Data collection method.............................................................................7
6. Reporting .................................................................................................8
7. Data processing......................................................................................10
8. Operational Results................................................................................12

Appendix A – email invitation ...................................................................13
Appendix B – questionnaire .......................................................................13
Appendix C – project personnel..................................................................36
Appendix D – who is Your Source? ............................................................37

# 1. Project scope and overview

The Scanlon Foundation in association with Monash University has conducted original primary research into social cohesion in Australia since 2007.

This project will provide information on social cohesion and immigration issues in Australian society. The project is designed to contribute to informed public discussion and planning in Australia.

This survey is being undertaken by researchers at Monash University.  This project aims to obtain the views of people who were born in Australia and whose parents were both born in Australia. It is concerned with views of Australian society and its future, with a focus on social cohesion and population issues.

Monash University has contracted Your Source to conduct the online version of this questionnaire.

The 15-minute questionnaire has been designed and optimised to an online survey format by Monash University and programmed by Your Source's dedicated programming team at Annik Systems.

The sample was sourced from OpinionsPaid™, Your Source's proprietary online panel.

Respondents were invited to participate online and invited directly via email.

Respondents were incentivised 300 Points (equivalent to $3.00 AUD) for their participation in this survey.

This methodology report will outline how the online survey was administered.

# 2. Timings

| Task | Completed |
|---|---|
| Research proposal submitted | 09/05/2014 |
| Project commissioned by Monash University | 27/05/2014 |
| Final project material provided by  Monash University | 04/06/2014 |
| Survey programming start | 06/06/2014 |
| Survey testing start | 11/06/2014 |
| Survey programming approved | 17/06/2014 |
| First day in field with Your Source panel | 17/06/2014 |
| Interim data check performed | 18/06/2014 |
| Your Source panel reminder emails sent | 14/07/2014 |
| Fieldwork completed and survey closed | 21/07/2014 |
| Raw SPSS data file check and delivery | 22/07/2014 |
| Weighted and coded SPSS data file delivery | 24/07/2014 |

# 3. Sample frame

The sample was drawn from the Colmar Brunton Group's proprietary online panel, OpinionsPaid™.

Since 1989 the Colmar Brunton Group have been building and creating a membership database for our research needs. The membership database is not sold; it is only used for the purpose of research conducted by the Clients of the Colmar Brunton Group. The online community OpinionsPaid™ was created in October 2004, with a web 2.0 community launched in 2005 with over 4300 survey being conducted and over 11200 mail outs from 2004 to 2013.

The Colmar Brunton Group has been actively managing its members since the company's inception in 1989 with the creation of the central location testing panel. The Community is actively managed by a team dedicated to providing quality research by quality respondents. With the creation of OpinionsPaid™ in 2004 the members from the central location testing panel were actively encouraged to join the online community. The members of OpinionsPaid™ have been recruited by multiple sources including but not limited to: Target invitations though panel partners, Telephone recruitment, Search engine optimization, Membership referrals, face to face or central location testing, print advertisements, targeted invitations to members of specialist managed marketing lists & river sampling through social networking sites.

OpinionsPaid™ members are solely used for market research purposes and only for research conducted by the Colmar Brunton Group, which Your Source is part of.

To become an OpinionsPaid™ member, Your Source have to be provided with a minimum of: Full name, residential address, Gender and Date of birth. Once someone joins OpinionsPaid™ they are directed to complete their full profile information. Which include over 70 questions across the following areas: advanced demographics, employment, business (for those who qualify), education, grocery purchasing behaviour, automotive, media usage, technology usage and ownership, Alcohol, tobacco, travel, finance and attitude.

The main incentive which is used at OpinionsPaid™ is a points system. The Points are accrued and can be used to purchase vouchers or allocated to a number of partner charities. The amount of Points a members can earn from a project is based the length and complexity. Being a customised research company every research project is assessed for how many Points should be paid to the members for the project at the proposal stage. If a project is piloted and it is found to be more complex or longer then quoted the Points are adjusted accordingly.

On this occasion, Your Source offered $3.00 worth of points to its panel members for completing the questionnaire, and $0.20 to members who did not qualify.

# 4. Sample size

The target for the project was to achieve n=1000 completed questionnaire with respondents aged 18 and over, who were born in Australia and whose parents were both born in Australia.

No specific quotas were given by Monash University, however, Your Source did set upper limits for gender, age (nested with gender) as well as location (not interlocking with gender or age).

The aim was to achieve a fairly homogenous sample, somewhat representative of the overall Australian population aged 18 and over.

The quotas were set as follows:

Table 1:

| Age / gender | Male | Female |
|---|---|---|
| 18-24 | 70 | 70 |
| 25-34 | 100 | 100 |
| 35-44 | 90 | 90 |
| 45-54 | 90 | 90 |
| 55-64 | 80 | 80 |
| 65-74 | 50 | 50 |
| 75+ | 50 | 70 |
| **Total** | **530** | **550** |

Table 2:

| | |
|---|---|
| NSW | 320 |
| VIC | 250 |
| QLD | 200 |
| SA | 75 |
| WA | 110 |
| TAS | 20 |
| NT | 10 |
| ACT | 20 |
| Other | 999 |

OpinionsPaid™ members were invited to participate in the survey via email.

The questionnaire included four screening questions to verify that participants met the three qualifying criteria:

- born in Australia
- both born in Australia
- at least 18 years of age
- residing in Australia

The n=1000 target was exceeded on the 1st July 2014. At the is stage, it was felt by the researcher that the final sampling frame did not include enough young males aged 18-24 and 25-34 for the analysis before weighting the survey data.

Your Source having just completed registered more members for its OpinionsPaid™ panel on the back of its Weekly Online Poll went back to field on the 14th July 2014. This included inviting new members as well as sending a reminder email to non-completers.

The survey was closed a week later on the 21st July 2014. A total of n=1093 participants completed the survey.

Attempts were made to keep the responses as close to the quota as possible and the survey link was closed to further participants once the quota was reached.

# 5. Data collection method

OpinionsPaid™ members were sent an email invitation to take part in the survey.

A template HTML invite is used for the majority of The Colmar Brunton group's projects but this can be tailored to our clients' needs as long as the invitation still complies with the ESOMAR, AMSRS and SPAM ACT 2003 regulations.

For members who require plain text we also send out plain text invitations in a standard format.

Using HTML and Plain text allows for maximum penetration to our members.

The Invitation advised the average length of the survey, the incentive they will receive on completion or termination, when the survey will be closed and the basic topic of the survey.

For the first day in field, a batch of 2000 members was selected, so as to be fairly representative of the general population of Australia aged 18 and over.

A copy of the invitation from the Your Source panel can be viewed in Appendix A.

The survey consisted of 52 questions; all closed questions besides the 'other specify' options given in some code frames.

The questionnaire design also included a number of grids. Monash University researchers made some changes to the initial version of the survey so as to accommodate the display limitations of an online survey setting.

Scripting logic also asked all respondents to answer all screening questions before exiting the survey. That way, we avoided giving away the screening criteria, which can easily be communicated in public internet forums that neither Monash University nor Your Source are either aware of, or able to monitor.

Your Source also made some changes to questions H1, H2 and H3. The scales were reversed to be consistent with the rest of the survey and not induce respondents in error.

A copy of the final version of the survey can be viewed in Appendix B.

The average survey length was anticipated to be a maximum of 15 minutes. The median survey length ended up being 13 minutes and 0 seconds.

A total of 56.890 email invitation and reminder emails were sent to take part in the survey.

# 6. Reporting

Progress reports were provided in Excel form every few days and on an ad hoc basis as requested by the researchers.

Updates included the amount of completed surveys and other terminated sample numbers, either as a result of not meeting the qualifying criteria (screened) or due to the quote target being reached (quota full).

Quota details for age, gender and location were also shown in the reports.

The first report also indicated:

- the actual incidence recorded against the quoted one; as well as
- the actual median survey length compared to the one budgeted at the proposal stage.

Whilst the incidence is automatically calculated by the Excel template, the median survey length remained the same on all the updates.

The final report looked as follows:

**Comments:** Another 5 completes from males aged 25-34. We're closing the survey and exporting the data today.

| Interview Progress | Total |
|---|---|
| Complete | 1093 |
| Incomplete | 201 |
| Screened | 1429 |
| QuotaFull | 1669 |
| Error | 0 |
| **Total interviews started** | **4392** |

| | |
|---|---|
| **Actual incidence:** | **43%** |
| Quoted incidence | 30% |
| **Actual median survey length:** | **13 minutes and 41 seconds** |
| Quoted median survey length: | 15 minutes |

| Unknown postcodes | Limit | Count | Rem. |
|---|---|---|---|
| Other | 999 | 4 | 995 |

| Location | Limit | Count | Rem. |
|---|---|---|---|
| NSW | 320 | 372 | 0 |
| VIC | 250 | 296 | 0 |
| QLD | 200 | 227 | 0 |
| SA | 75 | 92 | 0 |

| | | | |
|---|---|---|---|
| WA | 110 | 51 | 59 |
| TAS | 20 | 28 | 0 |
| NT | 10 | 3 | 7 |
| ACT | 20 | 20 | 0 |

| **Age** | **Gender** | **Limit** | **Count** | **Rem.** |
|---|---|---|---|---|
| 18 - 24 years | Male | 70 | 51 | 19 |
| 18 - 24 years | Female | 70 | 70 | 0 |
| 25 - 34 years | Male | 100 | 140 | 0 |
| 25 - 34 years | Female | 100 | 103 | 0 |
| 35 - 44 years | Male | 90 | 93 | 0 |
| 35 - 44 years | Female | 90 | 90 | 0 |
| 45 – 54 years | Male | 90 | 92 | 0 |
| 45 – 54 years | Female | 90 | 91 | 0 |
| 55 – 64 years | Male | 80 | 94 | 0 |
| 55 – 64 years | Female | 80 | 83 | 0 |
| 65 – 74 years, or | Male | 50 | 72 | 0 |
| 65 – 74 years, or | Female | 50 | 59 | 0 |
| 75 + years | Male | 50 | 30 | 20 |
| 75 + years | Female | 70 | 25 | 45 |

# 7. Data processing

Practices to ensure high quality data commenced well in advance of the raw data file being ready for processing. The following processes were followed to ensure the highest quality data was gathered for analysis purposes:

- During the programming stage, the survey was tested by multiple members of the project team from Your Source and Monash University until full satisfaction of the researchers.
- A full data checking and validation process was undertaken against the final questionnaire once a sufficient number of participants had completed the survey to ensure that unsuitable data was not included in future interim and final data exports. Once checking and validation was finalised the survey was launched fully.
- Final data was cross-checked and verified to ensure data of dubious quality (nonsensical verbatim answers or respondents straight lining tables and grids) was deleted prior to delivery to Monash University. 23 cases were deleted at this stage.
- Interim and final raw data files were supplied in SPSS .sav format. These files included responses to all completed survey questions
- The weighting was based on 2011 census data with three levels consisting of age, gender and location. Below is a summary of the factors in a matrix containing the 3 levels.

Table 3:

| | | | |
|---|---|---|---|
| Male | Sydney, Other NSW, Canberra, Other ACT | 18 - 24 | 1.68678045 |
| | | 25 - 34 | 0.51519081 |
| | | 35 - 44 | 0.73598692 |
| | | 45 - 54 | 1.32418089 |
| | | 55 - 64 | 1.35754728 |
| | | 65 - 100 | 0.79964174 |
| | Melbourne, Other VIC, Hobart, Other Tas | 18 - 24 | 0.96585379 |
| | | 25 - 34 | 0.68815754 |
| | | 35 - 44 | 1.32254716 |
| | | 45 - 54 | 0.76627472 |
| | | 55 - 64 | 0.65088716 |
| | | 65 - 100 | 1.03207476 |
| | Brisbane, Other QLD | 18 - 24 | 1.02787239 |
| | | 25 - 34 | 0.93970901 |
| | | 35 - 44 | 1.40621764 |
| | | 45 - 54 | 0.89485888 |
| | | 55 - 64 | 0.89403245 |
| | | 65 - 100 | 0.86103994 |
| | Adelaide, Other SA, Darwin, Other NT | 18 - 24 | 1.12895519 |
| | | 25 - 34 | 0.77486157 |
| | | 35 - 44 | 1.33872638 |
| | | 45 - 54 | 1.35791105 |
| | | 55 - 64 | 0.39095086 |
| | | 65 - 100 | 0.49360609 |
| | Perth, Other WA | 18 - 24 | 4.09353649 |
| | | 25 - 34 | 4.09353649 |

| | | | |
|---|---|---|---|
| | | 35 - 44 | 4.09353649 |
| | | 45 - 54 | 1.43884117 |
| | | 55 - 64 | 1.64409613 |
| | | 65 - 100 | 4.12540835 |
| Female | Sydney, Other NSW, Canberra, Other ACT | 18 - 24 | 0.92304749 |
| | | 25 - 34 | 0.88695549 |
| | | 35 - 44 | 0.91964762 |
| | | 45 - 54 | 1.26736766 |
| | | 55 - 64 | 0.82028614 |
| | | 65 - 100 | 1.69729138 |
| | Melbourne, Other VIC, Hobart, Other Tas | 18 - 24 | 0.73798737 |
| | | 25 - 34 | 1.11336331 |
| | | 35 - 44 | 1.47243445 |
| | | 45 - 54 | 0.71550835 |
| | | 55 - 64 | 0.93777809 |
| | | 65 - 100 | 1.1502159 |
| | Brisbane, Other QLD | 18 - 24 | 1.19637477 |
| | | 25 - 34 | 0.73935776 |
| | | 35 - 44 | 0.76044828 |
| | | 45 - 54 | 1.29828266 |
| | | 55 - 64 | 1.35671472 |
| | | 65 - 100 | 0.78461327 |
| | Adelaide, Other SA, Darwin, Other NT | 18 - 24 | 0.66723036 |
| | | 25 - 34 | 2.63880192 |
| | | 35 - 44 | 2.63880192 |
| | | 45 - 54 | 1.18163911 |
| | | 55 - 64 | 1.20356755 |
| | | 65 - 100 | 1.36821175 |
| | Perth, Other WA | 18 - 24 | 2.27381677 |
| | | 25 - 34 | 2.04625971 |
| | | 35 - 44 | 1.76266217 |
| | | 45 - 54 | 2.00087908 |
| | | 55 - 64 | 1.17389966 |
| | | 65 - 100 | 2.38951486 |

# 8. Operational Results

49,995 OpinionsPaid™ members were invited to take part into this survey. A reminder email was also sent 6,895 males aged 18-34 to achieve the final sample frame.

56,890 emails were sent in total for this study and a response rate of 7.37% was achieved. Response rate is calculated as total response (4,193) divided by total contacted (56,890).

Table 4 details the final outcomes (number and percentage) before the final data check and weighting process took place.

Table 4:

| Outcome | Count | % |
|---|---|---|
| Complete | 1093 | 25% |
| Incomplete | 198 | 5% |
| Quota full | 1671 | 38% |
| Screened | 1429 | 32% |
| **Total** | **4391** | **100%** |

The final incidence – complete/(complete+screened) – was 43%.

23 cases were removed in the final data check based on verbatim response in 'other specify' fields, 'straight liners' at B4, H1, H2 and H3, coupled with a survey length being shorter than the median value.

No cases were removed during the weighting process.

Table 5 shows the final sample frame after the final data check.

Table 5:

| | | NSW | VIC | QLD | SA | WA | TAS | NT | ACT | TOTAL |
|---|---|---|---|---|---|---|---|---|---|---|
| Male | 18 - 24 years | 11 | 17 | 13 | 5 | 0 | 2 | 0 | 2 | |
| | 25 - 34 years | 61 | 37 | 20 | 10 | 2 | 1 | 0 | 1 | |
| | 35 - 44 years | 41 | 15 | 14 | 6 | 5 | 5 | 0 | 3 | |
| | 45 – 54 years | 23 | 32 | 21 | 4 | 7 | 1 | 2 | 1 | |
| | 55 – 64 years | 18 | 30 | 18 | 18 | 5 | 3 | 0 | 2 | |
| | 65 – 74 years | 27 | 16 | 15 | 12 | 0 | 1 | 0 | 1 | |
| | 75 + years | 10 | 6 | 5 | 4 | 2 | 1 | 0 | 1 | |
| **Total Male** | | **191** | **153** | **106** | **59** | **21** | **14** | **2** | **11** | **557** |
| Female | 18 - 24 years | 21 | 23 | 11 | 8 | 3 | 1 | 0 | 2 | |
| | 25 - 34 years | 37 | 23 | 26 | 5 | 5 | 1 | 0 | 0 | |
| | 35 - 44 years | 36 | 16 | 27 | 1 | 6 | 3 | 0 | 1 | |
| | 45 – 54 years | 25 | 34 | 15 | 6 | 5 | 3 | 1 | 1 | |
| | 55 – 64 years | 32 | 24 | 12 | 6 | 7 | 0 | 0 | 2 | |
| | 65 – 74 years | 15 | 15 | 17 | 4 | 1 | 4 | 0 | 3 | |
| | 75 + years | 4 | 5 | 8 | 3 | 3 | 2 | 0 | 0 | |
| **Total Female** | | **170** | **140** | **116** | **33** | **30** | **14** | **1** | **9** | **513** |
| **TOTAL** | | **361** | **293** | **222** | **92** | **51** | **28** | **3** | **20** | **1070** |

# Appendix A – email invitation

Subject: Take part in the COHESION survey for 300 Points

FIND US ON FACEBOOK    FOLLOW US ON TWITTER

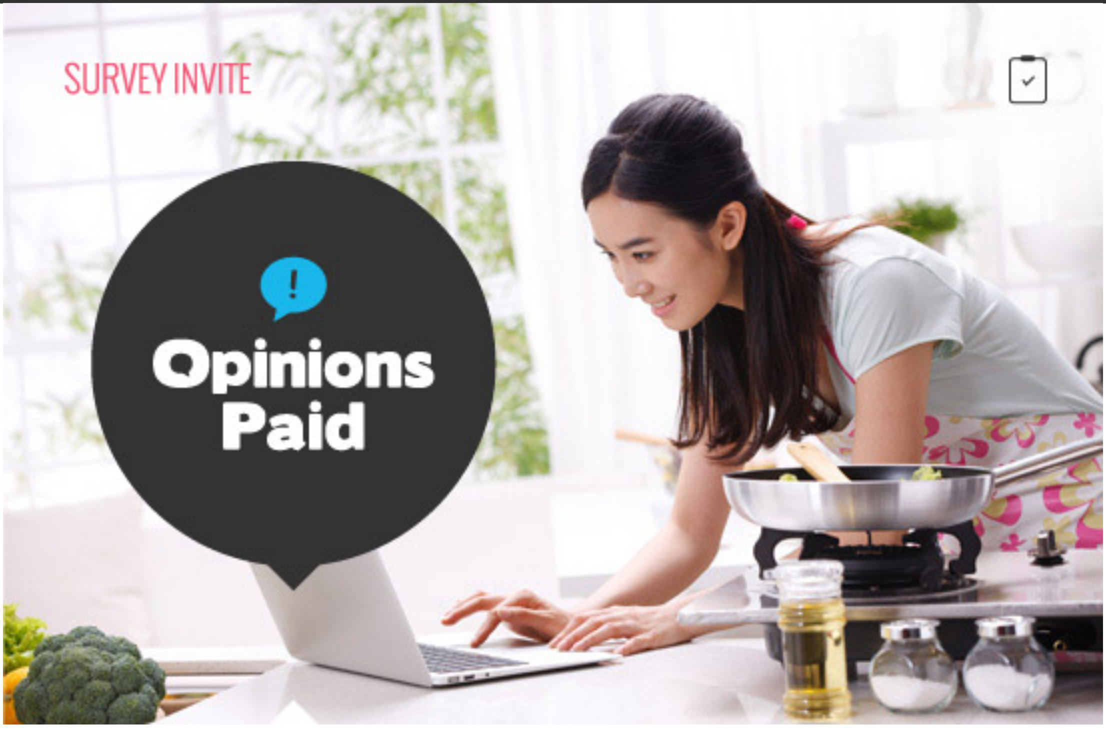


## COHESION SURVEY

Dear Laura,

Welcome to the COHESION survey, which is about issues Australians face nowadays.

Please click here or above right to start the survey.

Be assured that your personal details will not be stored in conjunction with your responses to any survey. This research is being done on behalf of Your Source.

START SURVEY

Survey details

Points: 300
if you qualify
or 20 if you don't.

Time: 15 minutes

Due date:
23/06/2014

Points will be credited by the 27/06/2014.

---

You have received this email because you signed up to become a member of OpinionsPaid or through Your Source.

If you no longer wish to be a member and participate in surveys, you can unsubscribe by emailing Unsubscribe. We thank you for your time and appreciate your opinions.

© 2014 OpinionsPaid

Powered by Your Source

# Appendix B – questionnaire

**Monash University**
**Social Cohesion Research Program**

**2014 ONLINE SURVEY**

**This survey is for persons who are aged 18 or over and**

**[A] Were born in Australia**

**AND**

**[B] Have both of their parents born in Australia**

**Hard quota:**

Total n=1000

**Soft quotas:**

| Age / gender | Male | Female |
|---|---|---|
| 18-24 | 70 | 70 |
| 25-34 | 100 | 100 |
| 35-44 | 90 | 90 |
| 45-54 | 90 | 90 |
| 55-64 | 80 | 80 |
| 65-74 | 50 | 50 |
| 75+ | 50 | 70 |

| | |
|---|---|
| NSW | 320 |
| VIC | 250 |
| QLD | 200 |
| SA | 75 |
| WA | 110 |
| TAS | 20 |
| NT | 10 |
| ACT | 20 |
| Other | 999 |

**DEM15 COUNTRY**
In which country were you and your parents born? **SR PER ROW**

| | AUSTRALIA | ANOTHER COUNTRY |
|---|---|---|
| a) Yourself | 1 | 2 |
| b) Your mother | 1 | 2 |
| c) Your father | 1 | 2 |

**DEM1a AGE**
Which age group you in? **SR**

1. Under 18 years
2. 18 - 24 years
3. 25 - 34 years
4. 35 - 44 years
5. 45 – 54 years
6. 55 – 64 years
7. 65 – 74 years, or
8. 75 + years
9. Prefer not to answer

**DEM2 GENDER**`VAR in DATA SET: gender`
Are you…? **SR**

1 Male
2 Female

**DEM18 POSTCODE**
What is your postcode? **SR / RANGE 200-9944**

3 _ _ _ _
4 Don't know
5 Prefer not to answer

**TERMINATE:**
**IF DEM15(a)=2 OR DEM15(b)=2 OR DEM15(c)=2**
**OR**
**IF DEM1a=1 OR 9**
**OR**
**IF DEM18=2 OR 3**

**TERMINATION TEXT**
Thank you for your participation. This is all we needed to ask you for today. We will contact you again shortly for another survey.

**AN1 PROBLEM**
To start with, what do you think is the most important problem facing Australia today? **SR**

1. Aboriginal / Indigenous issues (health, poverty, treatment, etc)
2. Asylum Seekers - poor treatment /refugees / boat people /illegal immigrants (sympathetic comment)
3. Asylum Seekers - too many /refugees / boat people /illegal immigrants (negative comment)
4. Crime/ law and order
5. Defense/National security/Terrorism
6. Economic issues - Economy/Unemployment/Jobs/Deficit/poverty
7. Education/ schools
8. Environment/ climate change/ water shortages (concern)
9. Environment - over-reaction to climate change/carbon tax (skeptical)
10. Government/ quality of/ politicians
11. Health/ medical/ hospitals
12. Housing shortages/ affordability/ interest rates
13. Immigration/population - too high, overcrowding /wrong people coming (negative)
14. Immigration/population - too low/ need more people (supportive)
15. Industrial relations/Trade unions
16. Racism
17. Social Issues - drug use, family breakdown, internet overuse, childcare
18. Women's issues (e.g.: equal pay/opportunity, violence, etc)
19. Other
20. Nothing
21. Don't know
22. Prefer not to answer

**A1 INCOME**
To what extent do you agree or disagree with the following statements?

| SR PER ROW | Strongly agree | Agree | Neither agree nor disagree | Disagree | Strongly disagree | None of the above/ Don't know | Prefer not to answer |
|---|---|---|---|---|---|---|---|
| a) People living on low incomes in Australia receive enough financial support from the government | 1 | 2 | 3 | 4 | 5 | 6 | 7 |
| b) In Australia today, the gap between those with high incomes and those with low incomes is too large. | 1 | 2 | 3 | 4 | 5 | 6 | 7 |
| c) Australia is a land of economic opportunity where in the long run, hard work brings a better life. | 1 | 2 | 3 | 4 | 5 | 6 | 7 |

**A5 FINANCIAL CIRCUMSTANCES**
Now a question about your own financial circumstances.

How satisfied or dissatisfied are you with your present financial situation? **SR**

6 Very satisfied
7 Satisfied
8 Neither satisfied nor dissatisfied
9 Dissatisfied
10 Very dissatisfied
11 Don't know
12 Prefer not to answer

**A6 JOB**
How worried are you that you will lose your job in the next year or so. Would you say…? **SR**

1. Very worried
2. Worried
3. Neither worried nor not worried
4. A little worried
5. Not worried at all
6. Does not have a job/retired
7. Don't know
8. Prefer not to answer

**B4 POLITICAL ACTION**
Now some questions about different forms of political action people can take.

Which, if any, of the following you have done over the last three years or so?

| SR PER ROW | Yes | No | Don't Know | Prefer not to answer |
|---|---|---|---|---|
| 1. Voted in an election | 1 | 2 | 3 | 4 |
| 2. Signed a petition | 1 | 2 | 3 | 4 |
| 3. Written or spoken to a Federal or State Member of Parliament | 1 | 2 | 3 | 4 |
| 4. Joined a boycott of a product or company | 1 | 2 | 3 | 4 |
| 5. Attended a protest, march or demonstration | 1 | 2 | 3 | 4 |

**B6a GOVERNMENT**VAR in DATA SET: Rtrusgov
How often do you think the government in Canberra can be trusted to do the right thing for the Australian people? Would you say…? **SR**

13 Almost always
14 Most of the time
15 Only some of the time
16 Almost never
17 Don't know
18 Prefer not to answer

**B8 POLITICS**
How interested are you in politics? Would you say…? **SR**

1. Very interested
2. Somewhat interested
3. Neither interested nor disinterested
4. Not interested
5. Not at all interested
6. Don't know
7. Prefer not to answer

**B9 POLITICAL SYSTEMS**
The following question specifies three types of political systems. For each one, would you say it is a very good, fairly good, fairly bad or very bad way of governing Australia?

| **ROTATE STATEMENTS**<br>**SR PER ROW** | Very good | Fairly good | Neither good nor bad | Fairly bad | Very bad | Don't know | Prefer not to answer |
|---|---|---|---|---|---|---|---|
| A democracy, in which the members of parliament are chosen in an election | 1 | 2 | 3 | 4 | 5 | 6 | 7 |
| Having a strong leader who does not have to bother with parliament and elections | 1 | 2 | 3 | 4 | 5 | 6 | 7 |
| Having experts, not government, make decisions according to what they think is best for the country | 1 | 2 | 3 | 4 | 5 | 6 | 7 |

**B10 GOVERNMENT SYSTEM**
Would you say the system of government we have in Australia works fine as it is, needs minor change, needs major change, or should be replaced? **SR**

1. Works fine as it is
2. Needs minor change
3. Needs major change
4. Should be replaced
5. Don't know
6. Prefer not to answer

**B11 DEMOCRACY**
Do you agree or disagree with the following statement? **SR**

*'Democracy may have its problems, but it is still the best form of government.'*

1 Strongly agree
2 Agree
3 Neither agree nor disagree
4 Disagree
5 Strongly disagree
6 Don't know
7 Prefer not to answer

**C7 PRIDE** `VAR in DATA SET: Rbelongp`
To what extent do you take pride in the Australian way of life and culture? Would you say…? **SR**

19 To a great extent
20 To a moderate extent
21 Only slightly
22 Not at all
23 Don't know
24 Prefer not to answer

**C8 BELONGING** `VAR in DATA SET:Rbelong2`
And to what extent do you have a sense of belonging in Australia? Would you say…? **SR**

25 To a great extent
26 To a moderate extent
27 Only slightly
28 Not at all
29 Don't know
30 Prefer not to answer

**C9 MODERN WORLD** `VAR in DATA SET: Rbelong3c`
Do you agree or disagree with the following statement? **SR**

*"In the modern world, maintaining the Australian way of life and culture is important."*

31 Strongly agree
32 Agree
33 Neither agree nor disagree
34 Disagree
35 Strongly disagree
36 Don't know
37 Prefer not to answer

**C1 IMMIGRATION** `VAR in DATA SET: Rthreat5`
Now some questions about immigration.

What do you think of the number of immigrants accepted into Australia at present? Would you say it is…? **SR**

38 Too high
39 About right
40 Too low
41 No opinion/ don't know
42 Prefer not to answer

**C2 IMMIGRANTS** VAR in DATA SET: Rmultic1, Rmultic2, Rmultic3

Do you agree or disagree with the following statements?

| | SR PER ROW | Strongly agree | Agree | Neither agree nor disagree | Disagree | Strongly disagree | None of the above/ Don't know | Prefer not to answer |
|---|---|---|---|---|---|---|---|---|
| Rmultic1 | a) Accepting immigrants from many different countries makes Australia stronger | 1 | 2 | 3 | 4 | 5 | 6 | 7 |
| Rmultic2 | b) Ethnic minorities in Australia should be given Australian government assistance to maintain their customs and traditions | 1 | 2 | 3 | 4 | 5 | 6 | 7 |
| Rmultic3 | c) Multiculturalism has been good for Australia | 1 | 2 | 3 | 4 | 5 | 6 | 7 |

**CN3 RESIDENTS** VAR in DATA SET: threat1, threat2, threat3, threat4

Do you feel positive, negative or neutral about the following categories of people coming to live in Australia as permanent or long-term residents?

| | SR PER ROW | Very positive | Somewhat positive | Neutral | Somewhat negative | Very negative | None of the above/ Don't know | Prefer not to answer |
|---|---|---|---|---|---|---|---|---|
| threat1 | a) Skilled workers (e.g Doctors or Nurses, plumbers etc) | 1 | 2 | 3 | 4 | 5 | 6 | 7 |
| threat2 | b) Those who have close family living in Australia (i.e. parents or children) | 1 | 2 | 3 | 4 | 5 | 6 | 7 |
| threat3 | c) Refugees who have been assessed overseas and found to be victims of persecution and in need of help | 1 | 2 | 3 | 4 | 5 | 6 | 7 |
| threat4 | d) Young people who want to study in Australia | 1 | 2 | 3 | 4 | 5 | 6 | 7 |

**CN7 ATTITUDE**VAR in DATA SET: Rmultic4prejud
Is your personal attitude positive, negative, or neutral towards…?

| **SR PER ROW** | Very positive | Somewhat positive | Neutral | Somewhat negative | Very negative | None of the above/ Don't know | Prefer not to answer |
|---|---|---|---|---|---|---|---|
| Christians | 1 | 2 | 3 | 4 | 5 | 6 | 7 |
| Buddhists | 1 | 2 | 3 | 4 | 5 | 6 | 7 |
| Muslims | 1 | 2 | 3 | 4 | 5 | 6 | 7 |

**CN5 ASYLUM SEEKEERS**
Which of the following four statements comes closest to your view about the best policy for dealing with asylum seekers who try to reach Australia by boat? SR

1. They should be allowed to apply for permanent residence
2. They should be allowed to apply for temporary residence only
3. They should be kept in detention until they can be sent back
4. Their boats should be turned back
5. Don't know
6. Prefer not to answer

Now thinking about any discrimination you may have personally experienced.

**D5 DISCRIMINATION**
Have you experienced discrimination because of your skin colour, ethnic origin or religion over the last 12 months? **SR**

1. Yes
2. No
3. Prefer not to answer

**ASK IF D5=1**
**D6 DISCRIMINATION FREQUENCY**
How often did you experience discrimination? Was it…? **SR**

1 Often – most weeks in the last year
2 About once a month in the last year
3 Three to six times in the last year
4 Just once or twice in the last year
5 Don't know
6 Prefer not to answer

**ASK IF D5=1**
**D7 DISCRIMINATION LOCATION**
Where have you experienced discrimination in the last year? Was it…?

| **SR PER ROW** | Yes | No | Don't Know | Prefer not to answer |
|---|---|---|---|---|
| 1 In your local area/ your neighbourhood | 1 | 2 | 3 | 4 |
| 2 On public transport | 1 | 2 | 3 | 4 |
| 3 In a shopping centre | 1 | 2 | 3 | 4 |
| 4 At a sporting event | 1 | 2 | 3 | 4 |
| 5 At your place of work | 1 | 2 | 3 | 4 |

**ASK IF D5=1**
**D7a DISCRIMINATION OTHER PLACE**
Have you experienced discrimination in another place we have not mentioned? **SR**

1. Yes (Please specify: _________)
2. No
3. Don't know
4. Prefer not to answer

**ASK IF D7a=1**
**D7b DISCRIMINATION OTHER PLACE**
And is there anywhere else? **SR**

1. Yes (Please specify: _________)
2. No
3. Don't know
4. Prefer not to answer

Next, your opinion on some more general issues.

**E1 PEOPLE** VAR in DATA SET: Trust_recgtrus
Generally speaking, would you say that most people can be trusted or that you can't be too careful in dealing with people? **SR**

43 Can be trusted
44 Can't be too careful
45 Can't choose/Don't know
46 Prefer not to answer

**E2 HAPPY** VAR in DATA SET: HappyRworth1
Taking ALL things into consideration, would you say that over the last year YOU have been…? **SR**

47 Very happy
48 Happy
49 Neither happy nor unhappy
50 Unhappy
51 Very unhappy
52 Don't know
53 Prefer not to answer

**E3 AUSTRALIA**
In three or four years, do you think that your life in Australia will be…? **SR**

54 Much improved
55 A little improved
56 The same as now
57 A little worse
58 Much worse
59 I don't think I'll be living in Australia
60 Cannot predict / Don't know
61 Prefer not to answer

MODULE F: NEIGHBOURHOOD AND VOLUNTARY WORK

And now thinking about your local area that is **within 15 to 20 minutes walking distance of where you live.**

**F2 LOCAL AREA** VAR in DATA SET: Rloccoh1, Rloccoh2, Rloccoh3
Do you agree or disagree with the following statements?

| | **SR PER ROW** | Strongly agree | Agree | Neither agree nor disagree | Disagree | Strongly disagree | There are not enough immigrants in my neighborhood to have any impact | Don't know | Prefer not to answer |
|---|---|---|---|---|---|---|---|---|---|
| Rloccoh1 | a) People in my local area are willing to help their neighbours? | 1 | 2 | 3 | 4 | 5 | 6 | 7 | 8 |
| Rloccoh2 | b) My local area is a place where people from different national or ethnic backgrounds get on well together | 1 | 2 | 3 | 4 | 5 | 6 | 7 | 8 |
| Rloccoh3 | c) I am able to have a real say on issues that are important to me in my local area. | 1 | 2 | 3 | 4 | 5 | 6 | 7 | 8 |

**F7 LOCAL AREA CHANGE** VAR in DATA SET: Rlocoh4area
Would you say that living in your local area is becoming better or worse, or is it unchanged? **SR**

1. Much better
2. Better
3. Unchanged
4. Worse
5. Much worse
6. Don't know
7. Prefer not to answer

The next two questions are about unpaid voluntary work. By this we mean any unpaid help you give to the community in which you live, or to an organisation or group to which you belong.

It could be to a school, a sporting club, the elderly, a religious group or people who have recently arrived to settle in Australia.

**B1 VOLUNTARY WORK**
Have you done any unpaid voluntary work of this kind in the last 12 months? **SR**

62 Yes
63 No
64 Don't know
65 Prefer not to answer

**ASK IF B1=1**
**B2new PARTICIPATE**VAR in DATA SET: RVolunt
How often do you participate in this sort of voluntary activity?  Is it…? **SR**

66 At least once a week
67 At least once a month
68 Three to four times a year
69 At least once a year
70 Less often than once a year
71 Don't know
72 Prefer not to answer

**F9b PERSONAL SAFETY**VAR in DATA SET: RSafety1
And now turning to your sense of personal safety.

How safe do you feel walking alone at night in your local area?  Would you say you feel…? **SR**

1. Very safe
2. Fairly safe
3. A bit unsafe
4. Very unsafe
5. Neither safe nor unsafe
6. Never walk alone at night
7. Don't know
8. Prefer not to answer

**F10 CRIME**VAR in DATA SET: RSafety2crime
Thinking about all types of crime in general, how worried are you about becoming a victim of crime in your local area? Would you say you are…? **SR**

1. Very worried
2. Fairly worried
3. Not very worried
4. Not at all worried
5. Don't know
6. Prefer not to answer

**F11 POLICE CONTACT**
Have you had any contact with the police in your local area in the last 12 months? **SR**

1. Yes
2. No
3. Don't know
4. Prefer not to answer

**ASK IF F11=1**
**F12 POLICE OPINION**
On the basis of your experience, do you agree or disagree that police…?

| SR PER ROW | Strongly agree | Agree | Neither agree nor disagree | Disagree | Strongly disagree | Don't know | Prefer not to answer |
|---|---|---|---|---|---|---|---|
| a) Treat people fairly and equally | 1 | 2 | 3 | 4 | 5 | 6 | 7 |
| b) Perform their job professionally | 1 | 2 | 3 | 4 | 5 | 6 | 7 |
| c) Are honest | 1 | 2 | 3 | 4 | 5 | 6 | 7 |

**ASK IF F11=1**
**F13 POLICE CONTACT**
How comfortable are you speaking with police? **SR**

1. Very comfortable
2. Comfortable
3. Neither comfortable/ not comfortable
4. Not comfortable
5. Not at all comfortable
6. Don't know
7. Prefer not to answer

**ASK IF F11=2 OR 3 OR 4**
**F14 POLICE IMPRESSION**
Is it your impression that the police?

| SR PER ROW | Strongly agree | Agree | Neither agree nor disagree | Disagree | Strongly disagree | Don't know | Prefer not to answer |
|---|---|---|---|---|---|---|---|
| d) Treat people fairly and equally | 1 | 2 | 3 | 4 | 5 | 6 | 7 |
| e) Perform their job professionally | 1 | 2 | 3 | 4 | 5 | 6 | 7 |
| f) Are honest | 1 | 2 | 3 | 4 | 5 | 6 | 7 |

**ASK IF F11=2 OR 3 OR 4**
**F15 POLICE COMFORT**
How comfortable would you feel speaking with police? **SR**

1. Very comfortable
2. Comfortable
3. Neither comfortable/ not comfortable
4. Not comfortable
5. Not at all comfortable
6. Don't know
7. Prefer not to answer

**F16 LAW COURTS**
Do you agree or disagree with the following statements about the law courts in Australia.

| SR PER ROW | Strongly agree | Agree | Neither agree nor disagree | Disagree | Strongly disagree | Don't know | Prefer not to answer |
|---|---|---|---|---|---|---|---|
| a) I trust the law courts in Australia | 1 | 2 | 3 | 4 | 5 | 6 | 7 |
| b) Australian law courts treat people with respect | 1 | 2 | 3 | 4 | 5 | 6 | 7 |

**MODULE H: PREDICTORS OF PREJUDICE**

**H1 AUSTRALIA**
Do you agree or disagree with the following statements about Australia?

| SR PER ROW | Strongly agree | Agree | Somewhat agree | Neither agree nor disagree | Somewhat disagree | Disagree | Strongly disagree |
|---|---|---|---|---|---|---|---|
| 1. I feel as if I belong to Australia | 1 | 2 | 3 | 4 | 5 | 6 | 7 |
| 2. When I discuss Australia I usually say 'we' rather than 'they' | 1 | 2 | 3 | 4 | 5 | 6 | 7 |
| 3. I identify with Australians | 1 | 2 | 3 | 4 | 5 | 6 | 7 |
| 4. I feel I am committed to Australia | 1 | 2 | 3 | 4 | 5 | 6 | 7 |
| 5. I feel a bond with Australians | 1 | 2 | 3 | 4 | 5 | 6 | 7 |
| 6. I see myself as Australian | 1 | 2 | 3 | 4 | 5 | 6 | 7 |

**H2 ETHNIC GROUPS**
Do you agree or disagree with the following statements about ethnic and cultural groups in Australia?

| **SR PER ROW** | Strongly agree | Agree | Somewhat agree | Neither agree nor disagree | Somewhat disagree | Disagree | Strongly disagree |
|---|---|---|---|---|---|---|---|
| 1. We should recognise that cultural and ethnic diversity is an important feature of Australian society | 1 | 2 | 3 | 4 | 5 | 6 | 7 |
| 2. A society that has a variety of ethnic and cultural groups is better able to tackle new problems as they occur | 1 | 2 | 3 | 4 | 5 | 6 | 7 |
| 3. We should do more to learn about the customs and heritage of different ethnic and cultural groups in this country | 1 | 2 | 3 | 4 | 5 | 6 | 7 |
| 4. It is best for Australia if all people forget their different ethnic and cultural backgrounds as soon as possible | 1 | 2 | 3 | 4 | 5 | 6 | 7 |
| 5. A society that has a variety of ethnic or cultural groups has more problems than societies with one or two basic cultural groups | 1 | 2 | 3 | 4 | 5 | 6 | 7 |
| 6. People who come to Australia should change their behaviour to be more like Australians | 1 | 2 | 3 | 4 | 5 | 6 | 7 |

**H3 CULTURES**
Do you agree or disagree with the following statements about people from other cultures?

| **SR PER ROW** | Strongly agree | Agree | Somewhat agree | Neither agree nor disagree | Somewhat disagree | Disagree | Strongly disagree |
|---|---|---|---|---|---|---|---|
| 1. I often spend time with people from other cultures | 1 | 2 | 3 | 4 | 5 | 6 | 7 |
| 2. I like meeting and getting to know people from other cultures | 1 | 2 | 3 | 4 | 5 | 6 | 7 |
| 3. I find it difficult to mix with people from other cultures | 1 | 2 | 3 | 4 | 5 | 6 | 7 |

**H4 FRIENDS** VAR in DATA SET: friends
Overall, approximately how many of your friends are from other cultures? **SR**

| | | | | | | | | | | |
|---|---|---|---|---|---|---|---|---|---|---|
| NONE | 1 | 2 | 3 | 4 | 5 | 6 | 7 | 8 | 9 | MORE THAN 10 |

**H5 BEST FRIENDS** VAR in DATA SET: bfriends
How many of your best friends are from other cultures? **SR**

| | | | | | | | | | | |
|---|---|---|---|---|---|---|---|---|---|---|
| NONE | 1 | 2 | 3 | 4 | 5 | 6 | 7 | 8 | 9 | MORE THAN 10 |

## DEMOGRAPHIC INFORMATION

We're nearly finished now. Just a final few questions to make sure we've spoken to a good range of people.

**DEM10 EDUCATION**`VAR in DATA SET: REduBin`
What is the highest level of education you have completed? **SR**

| | |
|---|---|
| 73 | Primary school |
| 74 | Year 7 to Year 9 |
| 75 | Year 10 |
| 76 | Year 11 |
| 77 | Year 12 |
| 78 | Trade/apprenticeship |
| 79 | Other TAFE/Technical Certificate |
| 80 | Diploma |
| 81 | Bachelor Degree |
| 82 | Post-Graduate Degree |
| 83 | Other (please specify: ______________) |
| 84 | Prefer not to answer |

**DEM11 EMPLOYMENT**`VAR in DATA SET: demempl`
Which one of these BEST describes your employment situation?  Are you …? **SR**

| | |
|---|---|
| 85 | Employed |
| 86 | Unemployed |
| 87 | Retired |
| 88 | Student |
| 89 | Home duties, or |
| 90 | Something else (please specify: ____________) |
| 91 | Don't know |
| 92 | Prefer not to answer |

**DEM13b FINANCES**`VAR in DATA SET: RIncome`
Which of the following terms best describes your financial circumstances today?  Would you say you are…? **SR**

| | |
|---|---|
| 93 | Prosperous |
| 94 | Living very comfortably |
| 95 | Living reasonably comfortably |
| 96 | Just getting along |
| 97 | Struggling to pay bills |
| 98 | Poor |
| 99 | Don't Know |
| 100 | Prefer not to answer |

**DEM17N RELIGION**
What is your religion, even if you are not currently practicing? **SR**

1 Catholic
2 Anglican (Church of England)
3 Uniting Church
4 Presbyterian
5 Greek Orthodox
6 Baptist
7 Lutheran
8 Islam
9 Buddhist
10 Judaism
11 Hinduism
12 Christian (no further information)
13 No religion
14 Other (please specify: ____________)
15 Don’t know
16 Prefer not to answer

**DEM22 VOTE**
Just one question about voting intentions. If there was a Federal election held today, for which party would you probably vote? **SR**

101 Labor Party
102 Liberal Party
103 National Party
104 Greens
105 Independents
106 Other (please specify: ____________)
107 Don’t Know
108 Prefer not to answer

NEXT SCREEN:

Dear Survey respondent

My name is Andrew Markus and I am a professor in the Faculty of Arts at Monash University. I am writing to provide more information about the survey that you have just completed. This survey is being undertaken by researchers at Monash University. This project aims to obtain the views of people who were born in Australia and whose parents were both born in Australia. **It is concerned with views of Australian society and its future, with a focus on social cohesion and population issues.**

**Why were you chosen to participate?**
Monash University has contracted *Your Source* to conduct the online version of this questionnaire. You have been selected because you have indicated your willingness to participate in *Your Source* surveys. Your answers to questions will be treated in the strictest confidence. Monash University will not receive any information from *Your Source* that could identify you or your household.

*Possible benefits*

This project will provide information on social cohesion and immigration issues in Australian society. The project is designed to contribute to informed public discussion and planning in Australia.

*Confidentiality*

Your responses to the survey questions are entirely anonymous.

*Storage of data*

Storage of the data will be undertaken under University regulations. The anonymous responses will be kept on secure computers on University premises for a minimum of five years.

**Use of data for other purposes**
Data resulting from the survey will be reported nationally and will be accessible to researchers.

*Results*

Once the project is completed the key findings will be accessible for a minimum of five years on the project website. The results of social cohesion surveys conducted between 2007 and 2013 are available at http://monash.edu/mapping-population/

**Thank you for your voluntary co-operation in this important survey. Your views are valuable and important in helping us to understand Australian society and its future development.**

Professor Andrew Markus
Monash University

**Please click Next to move on to the next screen**

**CLOSE**
Thank you again for completing this questionnaire. **Your views are valuable and important in helping us understand Australian society and its future development.**

This online survey was conducted by Your Source on behalf of Monash University researchers. Contact details with the university, should you require them, are

| If you would like to contact the **researchers** about any other aspect of this study, please contact: | If you have a **complaint** concerning the manner in which this research project (CF07/1240) is being conducted, please contact: |
|---|---|
| Dr Margaret Taft<br>Faculty of Arts,<br>Monash University, Clayton, Victoria<br>Tel: 03 9903 5018<br>Email: margaret.taft@monash.edu | Human Ethics Officer,<br>Monash Research Office,<br>First Floor, Building 3D<br>Monash University, Victoria 3800<br>Tel: 03 9905 5490<br>Email: muhrec@monash.edu |

# Appendix C – project personnel

Your Source always seeks to work in partnership arrangements with its clients, finding these to be the most satisfactory arrangement for both parties.  The following personnel will be associated with this project:

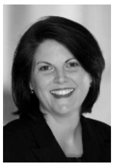

## Kylie Brosnan – Managing Director

Kylie joined Colmar Brunton in September 2009 her role to ensure a seamless interaction between clients, researchers and the fieldwork team on large scale quantitative projects.  Kylie has over 18 years research experience in market research operations management with specialist fieldwork suppliers and full service research houses.  She joins the Colmar Brunton team after 13 years with I-view, having spent her formative research years with AGB McNair in a field management and data administration role.

Kylie holds a Bachelor of Business (Operations Management and Marketing Majors) from the University of Southern QLD and a Diploma of Marketing Research from Charles Sturt University NSW.  She is a full member of the AMSRS and the current Chair for the Queensland Operations Research Group (AMSRS).
Kylie has great experience managing large scale quantitative studies including the South East Queensland Household Travel Survey, Translink Passenger counts and observational studies, TravelSmart Evaluation, International Visitors Survey, Queensland Visitors Survey, Longitudinal Study of Australian Children (Growing up in Australia), Stronger Families in Australia longitudinal study of the communities for children program.

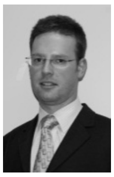

## Damien Marquez – Account Manager

Joining Your Source at the start of 2008, Damien is responsible for the management and implementation of primary Online Research projects.

Damien brings expertise to all stages of project development from initiation to completion.  With a penchant for achieving high levels of data quality through expert project management, Damien previously spent two years as a Manager at Foresearch, supplying pharmaceutical research data to the Australian market.

His experience includes a variety of qualitative and quantitative projects utilising Face-to-Face, Telephone, Online, Fax and Mail-out methods.

Before moving to Australia, Damien spent 5 years working in the very exacting field of multi-lingual Business-to-Business pharmaceutical and IT research.

# Appendix D – who is Your Source?

Your Source offers the highest quality data collection and analysis services to the Market Research industry through a focus on process control and consultation. This means excellent customer service, offering value for money.

Your Source proactively negotiates with its clients to ensure that operational planning is incorporated into project design.

As Australia's largest independently owned market research field agency, we offer great services with the capacity to manage your jobs, no matter what size they are. If you require analysis or reporting, we can do that too.

Most importantly, we create projects based on your requirements, as we actively seek long-term partnerships with our clients.

Your Source is an independently owned Australian Market Research company. We are the Australian Field members of WIN – the Worldwide Independent Network of Market Research agencies (http://www.winmr.com). WIN is an elite group of independent market and social research firms from across 21 countries (and growing), who collaborate to create a global platform in which international business can be conducted. This network is designed to not only share business and lead to new opportunities for us to work in Australia and abroad, but to share ideas, insights and products amongst some of the world's most savvy industry-leaders.

Your Source is a full-service agency, able to gather information via the traditional methods of Face-to-Face, Mail-out surveys and Telephone (CATI) as well as utilising newer technologies, such as online and hosted Web 2.0 Communities. We also host our own Market Research panel (which we do not on-sell for non-research purposes) of 100,000 Australian respondents, with their own community website (www.opinionspaid.com). While our offices are based on the eastern seaboard of Australia, we service the entire country and can facilitate F2F interviewing in rural communities and with specialist groups.

### CATI

We offer more than 120 Computer Assisted Telephone Interviewing (CATI) terminals across Australia, internet-enabled and equipped with market research-specific data capture software. Staff are highly trained and experienced in business-to-business, consumer and general surveys as well as more sensitive subject matter, for example domestic violence.

Our vast experience across industries as diverse as agriculture, finance, and telecommunications, through to luxury goods sets us apart from the competition. From opinion surveys to brand tracking to social research, we deliver.

### Online (CAWI)

Computer Assisted Web Interviewing (CAWI), commonly called 'Online', has changed the landscape of data collection in recent years, as it offers so many benefits to participants and clients. Firstly, of course, it's quick and easy for participants – a five-minute survey can include 25 or more questions. Secondly, it allows geographically diverse samples plus anonymity so we can survey those who would normally be difficult to recruit. Thirdly, the possibilities for surveys are very broad as technology evolves and can be fully customised to your research needs. It is perfect for consulting a sample group on an ongoing basis.

Your Source has broad experience in all levels of CAWI across social research, education, telecommunication, media, FMCG and electrical devices.

### Sensory Research

With the largest commercial sensory facilities in Australia and the highest level of accreditation (Grade 1 laboratories accredited through AQIS and ISO), you can be assured that the most important aspects of sensory research are second nature to Your Source.

We have incandescent lighting ensuring that food doesn't deteriorate, specifically trained staff, zoned air conditioning with positive pressure in the testing area and a host of other high-specification protocols, meaning better and more accurate testing. The facilities are suited to all age groups from pre-schoolers through to those with limited mobility. Stringent procedures are followed for storage, preparation and serving of samples. Over 11,000 product tests have been conducted by Your Source in Australia and New Zealand including FMCG, such as hot and cold food and drinks, cereals, canned food, cheese and washing powder.

### CLT (non-sensory) research

Central Location Testing – which might be face-to-face interviewing, shelf testing, intercept interviewing or usage simulation tests – can be run as projects on paper or laptop or even as in-home testing. This means we are able to run a project on- or off-site through a client-hosted interface.

All on-site respondents are audited for compliance before the study and briefed in a separate room. Our facilities are located next to or within large shopping centres, suited to all age groups and the research areas have adjoining viewing rooms with one way mirrors.

Your Source has experience across FMCG, government, telecommunications, luxury goods, IT and stationery.

### Qualitative research

Specialist qualitative interviewers, in both Melbourne and Sydney, and specialist rooms with closed-circuit television and viewing rooms (with one way mirrors), allow a great focus on the task at hand. We can provide you with a fully equipped private work space if you need it.

We pre-recruit respondents, and can sample in proportion to the Australian Bureau of Statistics for gender, education and qualification levels. We do not recruit from schools, clubs or groups, thus avoiding attitudinal bias. Respondents are not permitted to take part in research within a similar category on more than two occasions per year.

We can provide moderating, expert transcribing, note-taking and report writing. This can be done to whatever level our clients require. Our services also include trained hosting for groups and depth interviews, CD and DVD recording and the ability to host real-time online qual groups.

### DA / CADE

Your Source offers a range of analysis including:

- SAS & SPSS
- Card / Column
- ASCII

- Choice and Conjoint analysis
- Excel

We offer scripting for multi-language Online surveys, CATI and PDA.

Your data collection benefits from our skills in tracking, coding, data reformatting, digitising and analysis.

## YOUR SOURCE COMPLIANCE WITH MARKET & SOCIAL RESEARCH PRIVACY PRINCIPLES (MSRPP)

On 1st September 2003, the Market & Social Research Privacy Principles came in to effect. The MSRPP allows market research suppliers and buyers to conduct quality research whilst maintaining participant confidentiality. The MSRPP replaces the default National Privacy Principles, which restricts the ability of market researchers to conduct research. The MSRPP are only available to market research suppliers and buyers that are members of the Association of Market and Social Research Organisations (AMSRO). If a supplier or buyer is not a member, they must conduct research in accordance with the National Privacy Principles, which restricts market researchers from a variety of activities, particularly in the areas of customer lists, revealing the identification of the client, de-identification of data, and the use of sub-contractors.

Your Source's Quality Assurance Management Systems have been designed to conform to AS ISO 20252-2007. Quality assurance systems that apply to this project include:

> Recruitment,
> Data collection (face to face interviewing/ CATI interviewing/ online)
> Data Management and Processing (CATI/CAPI, hard copy data entry, data editing, data file management, coding, data analysis)
> Control of documents, data and records.
